# Plant-derived natural products and combination therapy in liver cancer

**DOI:** 10.3389/fonc.2023.1116532

**Published:** 2023-02-14

**Authors:** Yuqin Wang, Jinyao Li, Lijie Xia

**Affiliations:** Xinjiang Key Laboratory of Biological Resources and Genetic Engineering, College of Life Science and Technology, Xinjiang University, Urumqi, China

**Keywords:** plant-derived natural products, immunotherapy, combination therapy, liver cancer, molecular mechanisms

## Abstract

Liver cancer is one of the malignant cancers globally and seriously endangers human health because of its high morbidity and mortality. Plant-derived natural products have been evaluated as potential anticancer drugs due to low side effects and high anti-tumor efficacy. However, plant-derived natural products also have defects of poor solubility and cumbersome extraction process. In recent years, a growing numbers of plant derived natural products have been used in combination therapy of liver cancer with conventional chemotherapeutic agents, which has improved clinical efficacy through multiple mechanisms, including inhibition of tumor growth, induction of apoptosis, suppression of angiogenesis, enhancement of immunity, reversal of multiple drug resistance and reduction of side effects. The therapeutic effects and mechanisms of plant-derived natural products and combination therapy on liver cancer are reviewed to provide references for developing anti-liver-cancer strategies with high efficacy and low side effects.

## Introduction

Liver cancer is one of the most common malignant cancers with more than 906,000 new cases and 830,000 deaths annually worldwide (1). Liver cancer has brought significant threats and challenges to human’s physical and mental health as well as social and economic development (2). Currently, the main treatments for liver cancer include surgical resection (3), radiotherapy (4), chemotherapy (5), local ablation therapy (6) and liver transplantation (7). Although traditional therapies are effective for liver cancer at early stage, they have limited efficacy for advanced liver cancer due to severe side effect, drug resistance, multiple recurrences and metastases (8, 9). For the past few years, plant-derived natural products have been evaluated as potential anticancer drugs that preferentially kill tumor cells with low toxic effect on normal cells. A variety of plant-derived natural products with antitumor activities have been identified, such as alkaloids, terpenoids, phenols, flavonoids, which can inhibit tumor-cell invasion and migration (10), induce apoptosis (11), suppress angiogenesis (12) and proliferation (13). Although these plant-derived natural products have many advantages, such as abundant resources, low toxic effect and diversity in targets and molecular mechanisms, they have limited efficacy for clinical application due to low solubility and poor bioavailability (14). Therefore, there is an urgent need to develop new therapy with high efficacy and low side effects to prevent and treat liver cancer.

Numerous studies have shown that combined therapy has great potential value in treating of liver cancer. The combination of chemotherapy with natural compounds is likely to increase the efficacy of drug treatment as well as reduce the adverse responses (15). Some natural products may sensitize to conventional cytotoxic therapy, reinforce the drug effective concentration, reverse drug resistance, intensify the combined effect of both administered therapeutics or exert cytotoxic effects specifically on tumor cells (16). Moreover, natural products combined with immunotherapy, can reduce the development of cancer and enhance the immune function by targeting multiple signaling pathways (17). The desired effect could be to diminish burden on the patient’s organ by replacing part of the dose of a conventional chemotherapeutic with a natural substance with a defined effect. Many natural compounds are well tolerated by the patients and do not cause toxic effects even at high doses. Interaction of conventional chemotherapeutics with natural compounds introduces a new aspect in the research and therapy of cancer. It could be a complementing approach to potentially achieve improvements, while minimizing side effects associated with conventional chemotherapy. In this paper, we review the recent studies on the functions and mechanisms of plant-derived natural products and combination therapy in the treatment of liver cancer.

## Search strategy

**Figure 1:**
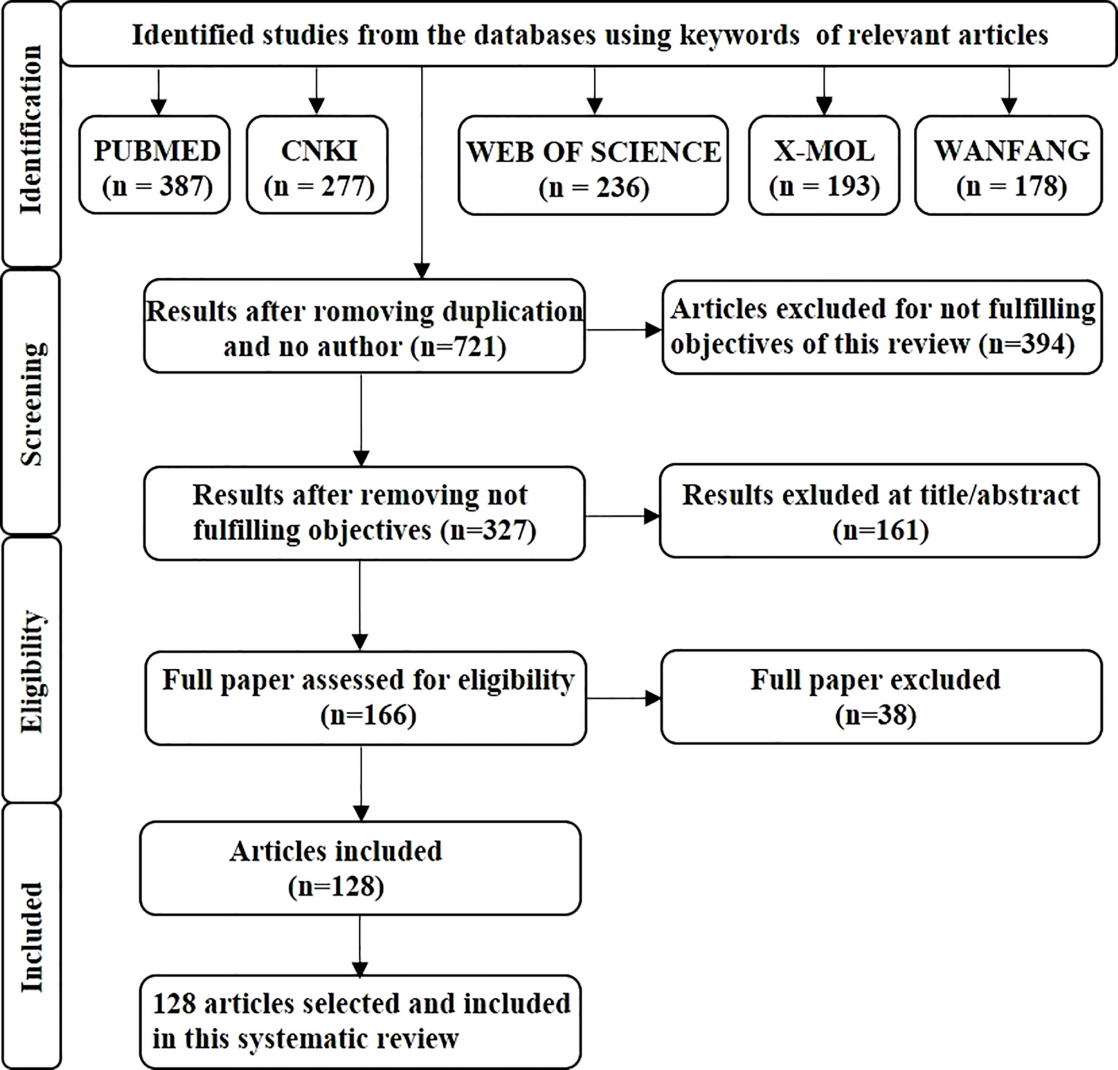
The flowchart provides an overview of the study search and selection process. A search of the databases PubMed, CNKI, Web of Science, X-MOL, and the Wanfang database was undertaken, and then duplicate articles and any which did not meet the criteria of our study were removed before the relevant articles were selected for further study.

This review followed the PRISMA guidelines. The primary search for article screening used in this review was conducted using PubMed (387), CNKI (277), Web of science (236), X-MOL (193) and the Wanfang database (178), and the medical subject headings (natural product, liver cancer, combination). After completing the initial search, therefore to avoid duplication, we removed 550 articles from the literature, and selected 721 articles. After screening for eligibility of the articles, 128 articles were included in the study. PRISMA flow chart indicates only the identification and screening processes of the anti-liver-cancer activity of plant-derived plant products and combination therapy (**Figure 1**).

## Plant-derived natural products in the treatment of liver cancer

### Alkaloids

Alkaloid is a kind of basic organic compounds containing nitrogen in plants. The representative anti-tumor compounds include matrine, berberine, evodiamine, camptothecin, vincristine and caffeine. Matrine exists in dried roots of *Sophora flavescent* and induces HepG2 cell apoptosis through increasing Bax/Bcl-2 ratio and inhibiting the ERK1/2 signaling pathway (18). Dai et al. reported that matrine inhibits BEL-7402 cell and SMMC-7721 cell invasion and metastasis by upregulating the expression of microRNA-199a-5p (miR-199a-5p) and reduces the levels of hypoxia-inducible factor-1α (HIF-1α) (19). Moreover, matrine also inhibits liver cancer migration and induces apoptosis through increasing the expression of microRNA-345-5p (miR-345-5p) and reduces the levels of circular RNA_0027345 (circ_0027345) and homeobox-containingD3 (HOXD3) (20). In addition, Zhang et al. researched that matrine at non-toxic dose could significantly suppress PLC/PRF/5 and MHCC97L cells migration and invasion. And, it also significantly decreased lung metastasis in orthotopic HCC mouse models (21). Berberine, an isoquinoline alkaloid, has been extracted from the rhizome of *Coptis* and *Phellodendri* and considered as anticancer drug in China because of its wide range of pharmacological effects. Berberine induced autophagy of HepG2 and MHCC97-L cells through activating Beclin-1 *via* enhancing p38 mitogen-activated protein kinase (MAPK) signaling pathway and inhibiting protein kinase B (Akt) activity (22). Hou et al. researched that berberine decreased the expression of CD147, thereby induced apoptosis of HepG2 and SMMC7721 cells in a dose-and time-dependent manner (23). In addition, berberine inhibits transforming growth factor-β/Smad (TGF-β/Smad) signaling pathway and interferes epithelial-mesenchymal transition (EMT), thereby inhibiting migration and invasion of HepG2 cells (24). A study showed that evodiamine induced apoptosis of HepG2 and PLHC-1 cells in a dose-dependent manner by inactivating phosphatidylinositol 3-kinase (PI3K) signaling pathway, downregulating B-cell lymphoma 2 (Bcl-2) expression and upregulating Bcl-2-associated X protein (Bax) (25). Evodiamine can also inhibit the proliferation of hepatocellular carcinoma cells by activating Hippo-Yes-associated protein signaling pathway *in vitro* and *in vivo* (26). Camptothecin has been used clinically to treat liver cancer, stomach and colorectal cancer for a long time, which can inhibit Huh7 and H22 cell proliferation, metastasis and angiogenesis by upregulating of reactive oxygen species (ROS) and nuclear factor E2-related factor 2 (Nrf2) expression, reducing the expression of N-cadherin, matrix metalloproteinase 9 (MMP9), Snail and Twist, and increasing E-cadherin (27). Notably, 10-hydroxycamptothecin (0.5 mg/L) inhibited the proliferation of BEL-7402 cells through induction of apoptosis, and showed synergistic effect with human cytokine-induced killer cells (optimal target ratio of 20:1) (28). According to reports, vincristine could decrease the expression of Ki-67, MMP2 and MMP9, and increase the expression of cleaved caspase 3, thereby inhibiting proliferation and reducing metastasis by PI3K/Akt signaling pathway in Hep3B cells (29). Caffeine is an alkaloids compound extracted from tea, cocoa bean and coffee fruit. Caffeine and X-ray irradiation have synergistic effect on the inhibition of cell proliferation and induction of apoptosis, and cell cycle arrest at G2/M phase (30). Cinchonine is a natural compound present in Cinchona bark. It exerts multidrug resistance reversal activity and synergistic apoptotic effect with paclitaxel in uterine sarcoma cells. Jin et al. demonstrated that cinchonine inhibited liver cancer cell proliferation and promoted apoptosis in a dose-dependent manner. It can activate caspase-3 and PARP1 cleavage, increased the expression of GRP78 and PERK, and suppressed HepG2 xenograft tumor growth in mice (31). As a transcription factor, the tumor suppressor p53 can participate in many important biological processes, such as cell cycle arrest and apoptosis. It can activate the expression of antioxidant proteins such as catalase, glutathione peroxidase 1 (GPX 1), manganese superoxide dismutase (MnSOD), which leads to the imbalance of ROS, and finally leads to apoptotic (32). Moreover, Sanguinarine significantly inhibited liver cancer cell proliferation in a p53-dependent manner by inducing cell cycle arrest and ROS-associated apoptosis. Importantly, it dramatically suppressed tumor growth in an HCC xenograft model, with little cytotoxicity (33).

### Terpenoids

Terpenoids are hydrocarbons and oxygenic derivatives in natural plants, which contain isoprene structure. It has been found that terpenoids have antitumor activities, such as saikosaponin D, triptolide, ginsenoside, artemisinin, celastrol and norcantharidin. Saikosaponin D is the most important active component of the Chinese medicinal herb *Bupleurum*, which can selectively up-regulate the protein expression of light chain 3II (LC3II) and Beclin 1, down-regulate the protein expression of P-S6K1, and induce autophagy by negatively regulating the mammalian target of rapamycin complex (mTORC) signaling pathway to reduce liver cancer cell growth (34). Moreover, it can increase radiation-induced apoptosis of liver cells *via* inhibiting mTOR phosphorylation to provide a possible potential therapeutic method for radio sensitization of liver cancer (35). In addition, Wu et al. discovered that saikosaponin D inhibited HepG2 cell proliferation by induction of apoptosis, improved the survival rate and suppressed the growth of liver tumors (36). Triptolide, a natural diterpenoid compound, is the main active ingredient of *Tripterygium wilfordii*, which inhibits the proliferation of HepG2 cells through induction of autophagy and apoptosis *via* up-regulating the expression of microRNA-194 (miR-194), LC3 and Beclin-1, and Bax/Bcl-2 ratio (37). Another study showed that it induced HepG2 cell apoptosis by inhibiting the expression of Ras (38). Gan et al. demonstrated that triptolide significantly suppressed the proliferation of H22 cells with IC_50_ value of 266.8 ng/mL at 24 h and induced apoptosis in H22 cells through decreasing the expression of cycloxygenase-2 (COX-2) (39). Ginsenoside-Rg3 inhibited cell proliferation and invasion of SMMC-7721 cells through PI3K/AKT pathway, which effectively decreased the levels of phosphorylated (p)-AKT, p-PI3K, MMP2, MMP9 and long non-coding RNA HOX antisense intergenic (lncRNA HOTAIR) (40). Recently, Zhang et al. showed that artemisinin enhanced the effect of anti-programmed cell death-ligand 1 (anti-PD-L1) immunotherapy by blocking the accumulation and function of myeloid-derived suppressor cells (MDSCs) *via* regulating PI3K/AKT/mTOR and MAPK signaling pathways and polarizing M2-like pro-tumor phenotype to M1-like anti-tumor phenotype (41). Dihydroartemisinine inhibits proliferation and induces apoptosis of HepG2 cells through regulating Bcl-2/Bax/caspase-3 apoptosis signal pathway (42). It can also induce liver cancer ferroptosis by promoting the formation of phosphatidylethanolamine-binding protein 1/15-Lipoxygenases (PEBP1/15-LO) and lipid peroxidation in cell membrane (43). Celastrol is a quinone methyl triterpene monomer compound of *T. wilfordii Hook. f.* and inhibits HepG2 cell proliferation by activating AMPK signaling pathway to suppress lipid metabolism and proliferation signal (44). Moreover, it can reduce the expression of nuclear factor kappa-B (NF-κB) and tumor necrosis factor alpha (TNF-α) in HepG2 and SMMC-7721 cells in a dose- and time-dependent manner (45). Recent studies have found that p53 can downregulate the expression of asparagine synthetase (ASNS) at the transcriptional level, thereby inhibiting asparagine synthesis and disrupting aspartate homeostasis, which can suppress the growth of lymphoma and colon tumors *in vivo* and *in vitro*. And depleting the levels of asparagine in cancer cells could release liver kinase B1 (LKB 1), and activated monophosphate-activated protein kinase (AMPK) -p53 signaling, triggered p21-dependent cell cycle arrest and promoted survival of cancer cells in asparagine-deficient conditions (46). Norcantharidin inhibits Huh7 cell proliferation and induces apoptosis in a time-and dose-dependent manner by activating p53 and p21 proteins and arresting cell cycle at G2/M phase (47). Tan et al. reported that genipin suppressed orthotopic HCC tumour growth through inhibiting IRE1a-mediated infiltration and priming of tumour associated macrophages (TAMs). It could reduce infiltration of inflammatory monocytes into liver and tumour, and inhibited the TAMs migration (48). Natural compound andrographolide (Andro), isolated from medicinal herb *Andrographis paniculata*, was reported to inhibit hepatoma tumor growth. It can inhibit the hepatoma tumor growth and alters the expression of miRNAs profile and downstream signals (49).

### Ployphenols

Polyphenol is a compound with multiple phenolic groups and includes curcumin, resveratrol, 6-gingerol, epigallocatechin gallate, and tannic acid. Curcumin is a spice and food coloring extracted from the rhizome of the perennial herb *turmeric* in Zingiberaceae. *In vitro* and *in vivo* results showed that curcumin downregulated the expression of Bcl2-associated transcription factor (BCLAF1), inhibited the activation of PI3K/AKT/glycogen synthase kinase-3β (GSK-3β) pathway, and triggered mitochondria-mediated apoptosis in liver cancer (50). Sun et al. reported that curcumin inhibited SMMC-7721 cell proliferation by induction of apoptosis *via* activating C/EBP homologous protein (CHOP) signaling pathway, reducing the expression of Bcl-2 and increasing cleaved caspase-3 and suppressed SMMC-7721 cell growth in xenograft tumor nude mice by activating endoplasmic reticulum (ER) stress pathway (51). Curcumin can induce liver cancer cell death by induction of apoptosis and pyroptosis. In addition, curcumin activates the gasdermin E (GSDME)-related scorch death and mitochondria-dependent apoptosis signaling pathways (52). Resveratrol is a phytoestrogen derived from grapes and berries and has several potential biological effects, including antioxidant, anti-inflammatory, cardioprotective and anticancer properties (53). Resveratrol inhibited the wound healing and invasion of HepG2 and Huh7 cells, increased the expression of E-cadherin, and decreased the expression of vimentin and Twist1. It inhibited metastasis and EMT through up-regulating the expression of microRNA-186-5p (miR-186-5p) (54). Furthermore, resveratrol can decrease the expression of membrane-associated RING-CH protein 1 (MARCH1) and p-Akt, increase the expression of phosphatase and tensin homolog deleted on chromosome 10 (PTEN) dose-dependently, hence inducing apoptosis and inhibiting the proliferation and invasion of HepG2 and Hep3B cells (55). 6-gingerol is an active polyphenolic compound extracted from ginger and can regulate the AKT/extracellular signal-regulated kinases 1/2 (ERK1/2) signaling pathway, increase P27/Kip1 and p21/Cip1 expression and inhibit cyclins D1, c-myc and cyclin-dependent kinase 4 (CDK4) protein expression, thereby arresting cell cycle at G2/M phase in Huh7 cells (56). Notably, a study showed that epigallocatechin gallate (EGCG) and EGCG derivative Y6 could significantly inhibit tumor growth and angiogenesis through MAPK/ERK1/2 and PI3K/AKT/HIF-1α/vascular endothelial factor (VEGF) pathways (57). Guo et al. demonstrated that EGCG combined with paclitaxel effectively inhibited the proliferation of HepG2 cells and the tumor growth in bearing cancer nude mice (58). In addition, it combined with adriamycin to exert synergistic anti-liver cancer effect, which induced apoptosis of BEL-7404/ADR cells and arrested cells in S/G2 phase of the cell cycle in a dose-dependent manner (59). Tannic acid is a natural polyphenol compound primarily found in fruits and nuts. Tannic acid can induce apoptosis by DNA disruption *via* caspase-dependent and caspase-independent mechanisms. It can also induce oxidative stress to promote liver cancer cell death (60). The combination of tannic acid with cisplatin induced HepG2 cell apoptosis through enhancing the activation of ER stress ATF6-CHOP pathway (61). Magnolol, a natural compound extracted from herbal plant *Magnolia officinalis*, has been recognized as a liver protection and antitumor drugs. It can significantly suppress tumor size and tumor growth rate, decreased the expression of p-ERK, NF-κB p65 (Ser536), MMP-9, VEGF, X-linked inhibitor of apoptosis protein (XIAP), and cyclinD1, and increased the expression of caspase-8 and caspase-9, which in turn induced apoptosis *in vivo* (62). Rosmarinic acid is a natural polyphenolic compound that exists in many medicinal species of Boraginaceae and Lamiaceae. Rosmarinic acid can regulated immune response and induced apoptosis of H22 cells. It could effectively inhibit the tumor growth through decreasing the expressions of IL-6, IL-10 and signal transducer and activator of transcription 3, up-regulating Bax, caspase-3 and down-regulating Bcl-2 (63).

### Flavonoids

Flavonoids are phenolic phytochemicals and are commonly found in fruits, vegetables and plant-based beverages (e.g., green tea and wine), which have a polyphenolic structure and are usually composed of a 15-carbon skeleton (including two benzene rings) and a heterocyclic nucleus containing an oxygen atom. Many studies have reported that flavonoids have a significant role in tumor treatment, such as kaempferol, silymarin, quercetin, chrysin and diosmetin. Kaempferol is a natural flavonol and widely distributes in many vegetables and fruits. Kaempferol remarkably decreased the expression of miR-21 and increased the expression of PTEN, thereby inhibiting HepG2 cell proliferation and metastasis through inactivating PI3K/AKT/mTOR signaling pathway (64). Moreover, it can promote apoptosis of HepG2 cells in a dose-dependent manner through decreasing the expression of CDK1 and increasing the expression of Bax and JUN (65). Another study showed that kaempferol was able to increase the protein expression of Bax, glucose-regulated protein of 78 kDa (GRP78), GRP94, PERK, cleaved ATF6, IRE1α, CHOP, caspase-3 and caspase-4, thereby inducing HepG2 cell apoptosis by mitochondrial pathway and ER stress pathway (66). Silymarin is a lipid compound extracted from the fruit of *Silybum marianum* and inhibits MHCC97 cell invasion and metastasis through reducing Akt phosphorylation and integrin β1, VEGF and MMP-9 expression. Moreover, it can restore the cell adhesion ability by upregulating E-cadherin, thereby inhibiting invasion and metastasis of liver cancer cells (67). Silibinin is another bioactive polyphenolic flavonoid isolated from the fruits and seeds of *S. marianum* (68), which can achieve anti-liver-cancer effects through multiple mechanisms, including induction of apoptosis, suppression of angiogenesis and block of cell cycle (69, 70). Quercetin is a dietary polyphenolic compound with wide distribution in onions, grapes, berries, apples, broccoli and cereals (71), and has the capability to reduce the expression of markers in cancer stem cells and myofibroblasts, as well as the expression of ABCC3 to reduce the acquisition of drug resistance in the early liver cancer stages (72). Quercetin inhibited the growth of HepG2 cells in a dose- and time-dependent manner, with IC_50_ values of 110.50, 63.73 and 46.38 µmol/L at 24 h, 48 h and 72 h, respectively, and suppressed the growth of subcutaneous xenografts in BALB/c mice (73). Another study showed that quercetin inhibited HepG2 cell proliferation by NF-κB pathway and activator protein-1 (AP-1)/c-Jun N-terminal kinase (JNK) pathway, which increased the expression of p-JNK, p-AKT, p-p38, caspase-3 and decreased the expression of Bcl-xL, p-ERK and NF-κB (74). Chrysin, also known as aspicin, is a natural flavonoid compound extracted from *Pterophylla globulina*, with extensive pharmacological activities (75). Chrysin significantly inhibited SMMC-7721 cell proliferation and induced apoptosis by activating MAPK signaling pathway (76). Moreover, chrysin could effectively inhibit the progression of tumor and promote the antitumor immunity of mice concomitant with increased CD4^+^ and CD8^+^ T cell proportions in tumor tissues of H22 xenograft mice model. Additionally, chrysin reduced the expression of PD-L1 through blocking of STAT3 and NF-κB pathways *in vitro* and *in vivo* (77). Diosmetin is an effective component of medicinal plants in southern China. Liu et al. found that diosmetin inhibited liver cancer cell metastasis, invasion and adhesion by regulating the PKCδ/MAPK/MMP pathway (78). It also can significantly downregulate the levels of Bcl-2, CDC2 and cyclinB1, and upregulate the levels of Bax, cleaved-caspase-3, cleaved-caspase-8, cleaved-PARP, Bak, p53 and p21, thereby inhibiting HepG2 cell proliferation and inducing cell apoptosis and arresting cell cycle at G2/M phase (79). Similarly, another study showed that diosmetin reduced the expression of Bcl-2, CDK1 and CCND1 in a dose-dependent manner *in vitro* and the expression of Ki67 *in vivo*, thereby inhibiting the proliferation and migration and arresting cell cycle at G1 phase of liver cells (80). Tan et al. observed that oral administration of baicalin completely blocked orthotopic growth of implanted HCC. And it induced repolarisation of TAM and M2-like macrophages, which is associated with elevated autophagy, and transcriptional activation of RelB/p52 pathway (81).

### Others

Emodin is an anthraquinone compound extracted from *Palmar rhubarb* and *Amomum amomatum* and inhibits liver cancer cell proliferation and metastasis by regulating VEGFR2-AKT-ERK1/2 signaling pathway and miR-34a signaling pathway (82). Aloin is a natural anthracycline compound, which can inhibit MHCC97H cell proliferation, migration and invasion and induce apoptosis through regulating Bcl-2/Bax ratio (83). Tanshinone is a phenoquinone derivative extracted from *Salvia miltiorrhiza* and inhibits HepG2 and Huh7 cell proliferation by blocking G0/G1 phase cell cycle *via* decreasing the expression of cyclin D1 and increasing p21, as well as inhibiting p53/damage-regulated autophagy and inducing apoptosis (84). As the most frequently mutated gene in human tumors, p53 can induce apoptosis, cell cycle arrest and cell senescence. And it plays an important role in suppressing proliferation of tumor, and is a major target for developing new drugs to reactivate its tumor suppressing activity for anticancer therapies. Taking p53 mutant Y220C as the core, Joseph et al. studied the structure-guided development of high-affinity small molecules stabilizing p53-Y220C *in vitro* and the synthetic route developed in the process. They found two new chemical probes with submicromolar binding affinity *in vitro*, which represents an important step toward the novel and effective Y220C ligand in the clinical evaluation of tumors (85). Cryptotanshinone significantly inhibited the cell viability of HepG2 cells with an IC_50_ of 93.73 μmol/L and induced ferroptosis by ROS accumulation *via* reducing glutathione (GSH) level and the expression of cystine/glutamate antiporter system light chain (xCT) and glutathione peroxidase 4 (GPX4) (86).

Natural polysaccharides are rich dietary components with significant roles in tumor prevention and treatment. Astragalus polysaccharides are the active component of the dried root of *Radix astragali* and can inhibit HepG2 cell proliferation (87), reverse the drug resistance of 5-fluorouracil (88), suppress EMT (89) and induce apoptosis (90). Astragalus polysaccharides can inhibit the growth of HepG2.215 cells by blocking HepG2.215 cells from entering the G2/M phase and inducing apoptosis (91). *Poria cocos* polysaccharides induced HepG2 cell apoptosis by inhibiting the expression of NOD-like receptor thermal protein domain associated protein 3 (NLRP3)/caspase-1/gasdermin D (GSDMD) in the classical pyroptosis pathway (92). Another study showed that *Lycium barbarum* polysaccharides inhibited SMMC-7721 cell proliferation and metastasis and induced apoptosis by reducing the expression of MMP-2, MMP-9 and VEGF (93). Saponins can significantly inhibit tumor cell proliferation. Astragaloside IV, also known as astragalus saponins, was able to upregulate the expression of caspase-3 and downregulate the expression of interleukin (IL)-6 by inhibiting the STAT3 signaling pathway, thereby inducing the apoptosis of liver cancer cells (94). Astragaloside IV also inhibits HepG2 cell proliferation and induces apoptosis by regulating oxidative stress and NF-κB signaling pathway (95). In addition, astragalus saponins promoted the secretion of IL-2 to enhance the proliferation and transformation of lymphocytes, thereby improving the immune function of rats and inhibiting liver cancer cells growth (96).

### Compounds

Zhu et al. investigated the influence of Huangqi Sijunzi decoction on 80 patients undergoing primary liver cancer operation. They found that Huangqi Sijunzi decoction can promote postoperative recovery of the liver function and immunologic function of the patients undergoing primary liver cancer operation, alleviate clinical symptoms and facilitate postoperative recovery of the patients (97). Yiqi Huoxue Fuzheng Jiedu detoxification is a compound with many kinds of traditional Chinese medicine, Jiang et al. explored the clinical effect of supplementing qi and activating blood circulation to assist the treatment of patients with advanced liver cancer. It is an effective method in adjuvant treatment of patients with advanced liver cancer, and it may be helpful to improve its clinical symptoms and liver function (98). Moreover, Zheng et al. researched that in the treatment of severe cancer pain due to primary liver cancer, external application of compound Gleditsiae Spina ointment has a marked analgesic effect, and can reduce the dose of opioid painkillers and the number of times of breakthrough pain and improve patients’ quality of life (99). Jing et al. observed the clinical effect of the therapy of Xingqi Sanjie Huayu prescription combined with reduced glutathione on 80 patients who had undergone surgery for primary liver cancer. It can effectively improve the clinical symptoms, reduce the levels of serum tumor markers, enhance the immune function and liver function, and reduce the incidence of adverse reactions (100). To investigate the clinical effect of Thymalfasin injection combined with Kanglaite injection and Chinese medicine Zhenggan prescription in the prevention of postoperative recurrence of primary liver cancer, chen et al. selected sixty patients with primary liver cancer. The triple therapy of Thymalfasin injection combined with Kanglaite injection and Chinese medicine Zhenggan prescription can significantly improve patients’ clinical syndrome and quality of life, promote the improvement of liver function, regulate immune function, reduce AFP level, has the advantages of low recurrence rate, has significant curative effect, and is worthy of further promotion and application (101). Furthermore, Zhao et al. founded that the therapy of Fuzheng Huayu Jiedu prescription can effectively improve the clinical effect on advanced liver cancer with less adverse reactions and high safety, and its mechanism may be related to its remarkable reduction of levels of inflammatory factors, inhibition of inflammatory responses, improvement of immune function and enhancement of resistance (102). Hedyotis diffusa injection is a Chinese patent medicine, which has the effect of clearing heat and detoxifying, benefiting dampness and swelling. Hedyotis diffusa injection combined with transarterial chemoembolization in the treatment of the patients with primary liver cancer can improve the disease control rate and improve the levels of T cell subsets. Moreover, it is superior to single transarterial chemoembolization (103).

**Supplementary Table 1** summarized the effects and mechanisms of plant-derived natural products on liver cancer. Although many plant-derived natural products are effective, they have limited efficacy for clinical application. Conventional mono-therapeutic methods target rapidly proliferating cells and destroy their growth, which it does not differentiate between healthy and cancerous cells. Chemotherapy can be toxic to the patient with multiple side effects and risks, such as nausea and vomiting and tumor metastasis (104). And it can also strongly reduce their immune system by affecting peripheral blood lymphocyte subsets and increasing susceptibility to host diseases (105). Although combination therapy can be toxic if one of the technologies is chemotherapeutic, the toxicity is remarkably less because a lower therapeutic dosage of each individual drug is demanded (106). It may be able to prevent the toxic effects on normal cells while concurrently producing cytotoxic effects on cancer cells. Further, monotherapy treatment is more develop drug resistance because continuous treatment with a single compound induces cancer cells to create alternative salvage approach (107). The most common mechanism of multidrug resistance is the elimination of drugs from the cell by ATP-dependent efflux pumps, thereby reducing intracellular drug concentrations and giving rise to resistance. As an example, cells in adenocarcinoma, when treated with adriamycin, upregulate the expression of ATP-binding cassette (ABC) transporter family, P-glycoprotein, to eliminate the drug, leading to a state of drug resistance (108). However, combination therapy also produced a more effective treatment response which target different pathways, and therefore this treatment modality reverses the drug resistance (109). Generally, conventional chemotherapy only targets non-stem cell tumor cells, resulting in an increasing proportion of stem cells in tumors after chemotherapy. And it is also a key mechanism for induction of drug resistance, regulation of metastasis and recurrence of malignant (110). However, combination therapy (for example quercetin in combination with chemotherapy drug doxorubicin) could reduce drug resistance and attenuated the likelihood of relapse (111). Therefore, combination therapy of plant-derived natural products with chemotherapeutics or immunotherapy is drawing researcher’s attention on liver cancer to induce apoptosis and inhibit cell proliferation and angiogenesis. It has the potential to develop therapeutic or adjuvant agents for human liver cancers.

## Anti-liver cancer mechanism by combination of plant-derived natural product ingredients

Mutations and epigenetic changes allow cancer cells to grow and replicate uncontrollably and invade normal tissues. This uncontrolled growth and proliferation arise due to loss of function mutations in tumour organelles such as lysosome (112), mitochondria (113), as well as endoplasmic reticulum (114). The limited efficiency and serious side effects associated with the use of conventional anticancer therapies encouraged scientists to focus on the discovery and development of new anticancer agents derived from natural products. For much of history, people have used plants and plant extracts to treat their ailments. Although the established natural plants as monotherapy of cancer had many advantages, they had limited efficacy for clinical application. If they are used in combination with other natural products, their anti-cancer effect is more effective. Secondary metabolites from plant sources like flavonoids, alkaloids, terpenoids, saponins, and others had been reported as important sources for potent anticancer agents. It was demonstrated that combination treatment acts by reducing the concentration of the compound required for drug efficacy through enhancing the effect on the same target or mechanism of action. The anticancer effect of these natural products in combination with others is mediated by different mechanisms, including inhibiting proliferation, blocking cell cycle and reversing multidrug resistance.

### Inhibit liver cancer cells proliferation

Proliferation and invasion of liver cancer cells were inhibited through decreasing the expression of the tumor suppressor gene deleted in liver cancer 1 (DLC1), translationally controlled tumor protein (TCTP) and Cdc42 (115). Resveratrol in combination with dihydroartemisinine acts by regulating DLC1/TCTP protein expression and inhibiting migration-related signaling pathways in HepG2 and MDA-AB231 cells through inducing Cdc42 regulation of JNK/NF-κB and N-WASP signaling pathways (116). PI3K-AKT signaling pathway is closely related to the proliferation of liver cancer cells (117). The combination of natural products acts by inducing anti-tumor effect through inhibiting the invasion and proliferation of tumor cells. Sodium arsenite and astrageoside IV inhibit HepG2 cell proliferation, block cell cycle, promote apoptosis, and suppress tumor growth. It can reduce the expression of PI3K, AKT, mTOR through PI3K/AKT/mTOR signaling effect, and the synergistic effect is more significant (118). Apoptosis an active and ordered multistep cell death process that controlled by genes, and involves two major pathways, including intrinsic and extrinsic apoptosis pathways in human cancer cells. The intrinsic pathway of apoptosis is mediated mainly by mitochondria within the cell, and this pathway is regulated by pro-apoptosis Bcl-2 family proteins (119). Natural products principally induced liver cancer cells apoptosis and inhibited proliferation through caspase cascade reaction and NF-κB signaling pathway.

Wen et al. developed citrus net skin black tea (CRPBT) by combining citrus reticulate peel and black tea. CRPBT can significantly down-regulate the phosphorylation of PI3K and AKT in liver cancer cells, up-regulate the Bax/Bcl-2 ratio, and inhibit the expression of MMP2/3, N-cadherin and Vimetin proteins. It can inhibit invasion, proliferation and induce apoptosis of liver cancer cells (120). A combination of curcuma zedoary and kelp could inhibit liver cancer cell proliferation and metastasis by inhibiting endogenous H2S production and down-regulating the transcription activator 3 (STAT3)/BCL-2 and VEGF pathway *in vitro* and *in vivo* (121). Bufalin (BFL) and cinobufagin (CBF) have a synergistic effect. Metabolic pathways, including methionine metabolism, energy metabolism, lipid metabolism, and amino acid metabolism, were modulated and subsequently led to apoptosis and cell cycle arrest of HepG2 cells. Zhang et al. researched that the cotreatment group can significantly decrease mitochondrial membrane potential (MMP) and oxygen consumption rate (OCR), which associated with maximal respiration (122). Triptolide and sodium cantharidinate exert a synergistic toxic effect on hepatoma cell line 7721, which is related to increasing capase-3 activity and suppression of NF-κB (123). Quercetin and rosmarinic acid had a more significant synergistic effect on inhibiting HepG2 cells proliferation and migration in a dose-dependent manner by up-regulating the expression of E-cadherin and down-regulating N-cadherin gene expression (124). Manikandan et al. showed that curcumin in combination with catechin which is polyphenolic compound have a synergistic effect on inhibiting the proliferation and inducing cell apoptosis. It treated HepG2 cells showed small morphological changes which significantly destruction of monolayer, and change the DNA fragmentation (125). Wang et al. evaluated the effects of co-treatment of vincristine and berberine on hepatic carcinoma cell lines. They find that combinational usage of these two drugs can significantly induce cell growth inhibition and apoptosis even under a concentration of vincristine barely showing cytotoxicity in the same cells when used alone (126). The combination of arsenic trioxide and tanshinone have synergistic effect, attenuation and antihepatocarcinoma effect. Tanshinone can reduce the blood routine changes and liver and kidney function damage caused by arsenic trioxide (127). Cryptotanshinone induced liver cancer cells apoptosis and enhanced the effect of Arsenic trioxide by downregulating phosphorylated STAT3, Bcl-2 *in vitro* and *in vivo* (128).

### Block liver cancer cells cycle

The cell cycle system plays a critical role in regulating activities such as cancer cell proliferation and apoptosis. The pathogenesis of neoplasms because of lost control in cycle progression is common. Uncontrol cell division is an essential factor in the development of cancer. The cell cycle consists of G1, the S-phase, G2, and the M (mitotic) phase (129). Cells are preparing for DNA synthesis during G1 and they perform surveillance to establish the integrity of newly synthesized DNA during G2 before initiating mitosis. Chromosomal DNA is replicated during the S-phase. If this phase arrest, the cells will undergo variation or abnormal division, which will then terminate the proliferative (130). In the G2 phase, after the nucleic acid is amplified, the other cellular components are partitioned between two daughter cells during the M-phase. If the stagnation occurs, the normal growth and gathering of the cells is hindered, which inhibit proliferation (131). Cyclins and their cognate cyclin-dependent protein kinases (CDKs) are necessary components required for traversing the cell division cycle, with controlling balance of all stages in cell cycle (132). CDK2/4/6, Cyclin D1, and Cyclin E are the major regulatory proteins in the G1 phase; and the reduction of Cyclin B1 and CDK1/2 activity is a key marker of cycle arrest in the G2/M phase (133, 134).

Zhang Di demonstrated that chrysin in combination with diosmetin or triptolide acted by the induction of apoptosis through inhibiting HepG2 cells migration as well as arresting the cell cycle in G1 and G2 phases *in vitro*. Moreover, it had a stronger inhibitory effect on H22 solid tumors than single drug application (135). Xiao et al. revealed that a combination of biochanin A with BRAF inhibitor SB590885 synergistically inhibiting proliferation by disrupting of the ERK MAPK and the PI3K/AKT pathways *in vitro*, and promoting cell cycle arrest and apoptosis (136). Quercetin in combination with maleic anhydride derivatives acts by inducing apoptosis of liver cancer cells through arresting cells in S-phase by oxidative stress response, causing DNA damageactivating the intrinsic apoptosis pathway (137). Curcumin (20 µmol/L) combined with vincristine (1 pg/L) inhibits proliferation and clone formation of HepG2 cells, reduces mitochondrial membrane potential and MDR1 and LRP protein expression, increases P21 expression, thereby blocking G2/M phase cell cycle and inducing apoptosis (138). Curcumin (5 pg/L) and glycyrrhetinic acid (10 pg/L) obviously inhibited HepG2 cells proliferation in a concentration- and time-dependent manner, arrested the G2 phase, and the synergistic effect is more remarkably (139). The combination of Curcumin (40 µmol/L) and artemisinin (80 µmol/L) could effectively inhibit HepG2 cells proliferation and arrest cell cycle and promote apoptosis. In addition, it can inhibit telomerase activity in liver cancer cells (140). Tanshinone IIA block subG1 phase cell cycle, while resveratrol arrest S phase and G2/M phase of HepG2 cells. The combination of them induce cell apoptosis and arrest subG1 cell cycle. Moreover, it exerted synergistic cytotoxicity and robustly induces apoptosis, and DNA fragmentation (141). Berberine (15 µmol/L) combined with tarpinine derivative HMQ1611 (15 µmol/L) inhibits cyclin D1, cyclin E, CDK1 expression and arrests the cell cycle at G1 phase through PI3K/AKT/mTOR signaling pathways to induce apoptosis of HepG2 cells. Furthermore, the combination therapy had strong suppressive effect on proliferation by inhibiting the phosphorylation of LDL receptor-associated protein, which was related to Wnt/β-catenin signaling pathway (142). Liu et al. showed that different concentrations of norcantraridin (12.5-200 µmol/L) and evodiamine (2.5-40 µmol/L) inhibited HepG2 cells growth and arrested G2/M phase cell cycle and induced apoptosis. Moreover, it has obvious synergistic inhibitory effect on anti-proliferation and pro-apoptosis through increasing the expression of Bax and decreasing the expression of Bcl-2 (143). Triptolide (0.01-5 pg/L), glycyrrhetinic acid (1-10 pg/L), rhein (1-10 pg/L) and paclitaxel (1-20 pg/L) inhibit HepG2 cell proliferation in a dose-dependent manner through blocking G2/M phase cell cycle. In addition, glycyrrhetinic acid, paclitaxel and rhein can enhance the inhibitory effect of low concentration of tripterine on HepG2 cell proliferation (144).

### Reverse liver cancer cells multidrug resistance

Liver cancer cells will be exposed to a certain chemotherapy drug for a long time under chemotherapy. And it can develop multidrug resistance (MDR) to this drug and cross-resistance to other chemotherapy drugs with different functional structures, leading to chemotherapy failure (145). This mechanism of multidrug resistance arises due to activity of the superfamily members of adenosine triphosphate binding cassette (ABC) such as P-glycoprotein (P-gP), multidrug resistance associated protein (MRP) and breast tumor resistance protein (BCRP) (146). Plant-derived natural products act by enhancing the tumor killing effect of chemotherapeutic drugs and inducing their anti-tumor activity through reversing the multidrug resistance of liver cancer cells.

Sun et al. demonstrated that curcumin reversed the multi-drug resistance caused by cucurbitacin B through inhibiting the expression of P-gP, thereby inducing liver cancer cell apoptosis (147). Shen et al. researched that coumarin derivatives, as an inhibitor of microtubule affinity-regulating kinase 4 (MARK4), induce apoptosis of liver cancer cell by increasing microtubule dynamics and the sensitivity of paclitaxel (148). The curcumin antiproliferative effect was confirmed by Nan et al., who reported marked induction apoptosis of liver cancer cells, reversed the drug resistance of paclitaxel through inhibiting NF-κB pathway stimulated Lin28B expression (149). Xu et al. prepared folate-PEG-mesoporous silica nanoparticles loaded with paclitaxel, which had a good targeting effect on liver cancer cells and enhanced the anti-tumor effect of paclitaxel (150). A combination of esculetin with paclitaxel acted by induce apoptosis of liver cancer cells through up-regulating Bax/Bcl, inducing the activation of caspase-8 and caspase-3, as well as ERK signal pathway (151). Jiang et al. identified the antitumor effects of resveratrol on HepG2 cell, in addition to up-regulate p53, Bax and other proteins, and reverse drug resistance of paclitaxel (152). The combination of emodin (10 mg/L) and curcumin (10 mg/L) enhances sensitivity of BEL-7402 hepatoma cells and reduces the dosage of emodin and induces apoptosis (153). Curcumin (5-20 µmol/L) and paclitaxel (0.05-0.2 µmol/L) have synergistic anti-proliferation and pro-apoptosis effects on Hep3B cells. It can decrease paclitaxel-induced NF-κB activation, mediate Lin28 level, thereby enhancing sensitivity of liver cancer cells to paclitaxel (154). Matrine also reverses resistance of K562/ADM cells to doxorubicin and vincristine by arresting cell cycle and inducing autophagy (155). **Table 1** summarizes their effects and mechanisms on liver cancer.

**Table 1:** Effect and mechanism by combination of plant-derived natural product ingredients on liver cancer.

Combination Therapy	Concentration	Cell Line/Model	Mechanism	Reference
Dihydroartemisinine/ Resveratrol	dihydroartemisinine: 25 μmol/L resveratrol: 50 μmol/L	HepG2 MDA-AB-231	Regulated DLC1/TCTP protein expression and induced Cdc42 regulation of JNK/NF-κB and N-WASP signaling pathways	(116)
Sodium arsenite/ Astrageoside IV	sodium arsenite: 0.5 μg/mL astrageoside IV: 0.8 μg/mL	HepG2	Reduced the expression of PI3K, AKT, mTOR through PI3K/AKT/mTOR signaling effect	(118)
Citrus reticulate peel/ Black tea	CRPBT: 0-1 mg/mL	HepG2 Bel 7402	Down-regulated the phosphorylation of PI3K and AKT, up-regulated the Bax/Bcl-2 ratio, and inhibited the expression of MMP2/3, N-cadherin and Vimetin proteins	(120)
Curcuma zedoary/ Kelp	curcuma zedoary: 4000 mg/kg kelp: 4000 mg/kg	H22	Inhibited endogenous H2S production and down-regulated the pSTAT3/BCL-2 and VEGF pathway *in vitro* and *in vivo*	(121)
Bufalin/ Cinobufagin	bufalin: 8 nmol/L cinobufagin: 50 nmol/L	HepG2	Decreased mitochondrial membrane potential (MMP) and oxygen consumption rate (OCR), which associated with maximal respiration	(122)
Triptolide/ Sodium cantharidinate	triptolide: 9-36 μg/mL sodium cantharidinate: 12-50 μg/mL	SMMC7721	Increased capase-3 activity and suppression of NF- κB	(123)
Quercetin/ rosmarinic acid	quercetin: 12.5-100 μmol/L rosmarinic acid:12.5-100μmol/L	HepG2	Up-regulated the expression of E-cadherin and down-regulated N-cadherin gene expression	(124)
Curcumin/ Catechin	curcumin: 50 μmol/L catechin: 25 μmol/L	HepG2	Inhibited the proliferation and induce cell apoptosis	(125)
Vincristine/ Berberine	vincristine: 0-4 nmol/L berberine: 0-40 nmol/L	HepG2 SMMC7721	Regulated the signals related to mitochondrial function, apoptotic pathway and endoplasmic reticulum stress	(126)
Arsenic trioxide/ Tanshinone	arsenic trioxide: 2.5 mg/kg tanshinone: 500 mg/kg	Bel-7404 nude mice	Reduced the blood routine changes and liver and kidney function damage caused by As2O3	(127)
Cryptotanshinone/ Arsenic trioxide	cryptotanshinone: 10,20 μmol/L arsenic trioxide: 1, 2 μmol/L	Bel-7404	Induced liver cancer cells apoptosis and enhanced the effect of Arsenic trioxide by downregulating phosphorylated STAT3, Bcl-2 *in vitro* and *in vivo*	(128)
Chrysin/ Diosmetin/ Triptolide	chrysin: 1.25-20 μmol/L diosmetin: 3.125-50 μmol/L triptolide: 0.78-12.5 nmol/L	HepG2 H22	Induced apoptosis and inhibited cell migration as well as arrested the cell cycle in G1 and G2 phases	(135)
Biochanin A/ SB590885	biochanin A: 75 μmol/L SB590885: 12 μmol/L	Bel-7402 SK-Hep-1	Inhibited proliferation by disrupting of the ERK MAPK and the PI3K/AKT pathways *in vitro*, and promoted cell cycle arrest and apoptosis	(136)
Quercetin/ Maleic anhydride derivatives	quercetin: 50 mmol/L maleic anhydride derivatives: 0.01 mmol/L	HuH7 HepG2	Induced apoptosisand arresting S-phase cell cycle by oxidative stress response, causing DNA damageactivating the intrinsic apoptosis pathway	(137)
Curcumin/ Vincristine	curcumin: 20 µmol/L vincristine: 1 pg/L	HepG2	Reduced mitochondrial membrane potential and MDR1 and LRP protein expression, increased P21 expression	(138)
Curcumin/ Glycyrrhetinic acid	curcumin: 5 pg/L glycyrrhetinic acid: 10 pg/L	HepG2	Inhibited the proliferation, promoted apoptosis and arrested the G2 phase in a concentration- and time-dependent manner	(139)
Curcumin/ Artemisinin	curcumin: 40 µmol/L artemisinin: 80 µmol/L	HepG2	Inhibited proliferation and telomerase activity and arrested cell cycle and promoted apoptosis	(140)
Tanshinone IIA/ Resveratrol	tanshinone IIA: 5 µg/mL resveratrol: 5 µg/mL	HepG2	Induced cell apoptosis and arrested subG1 cell cycle and DNA fragmentation	(141)
Berberine/ HMQ1611	berberine: 15 µmol/L HMQ1611: 15 µmol/L	HepG2 Bel-7402 SMMC-7721	Inhibited the phosphorylation of LDL receptor-associated protein, which is related to Wnt/β-catenin signaling pathway	(142)
Norcantraridin/ Evodiamine	norcantraridin: 12.5-200 µmol/L evodiamine: 2.5-40 µmol/L	HepG2	Increased the expression of Bax and decreased the expression of Bcl-2	(143)
Triptolide/ Glycyrrhetinic acid/ Rhein/ Paclitaxel	triptolide: 0.01-5 pg/L glycyrrhetinic acid: 1-10 pg/L rhein: 1-10 pg/L Paclitaxel: 1-20 pg/L	HepG2	Inhibited proliferation through block G2/M phase cell cycle	(144)
cucurbitacin B/ Curcumin	cucurbitacin B: 143.2 μmol/L curcumin: 108.6 μmol/L	BEL7402/5-Fu	Reversed the multi-drug resistance caused by terpenoid cysurin B through inhibiting the expression of P-gP, thereby inducing liver cancer cell apoptosis	(147)
Coumarinderivatives/ Paclitaxel	coumarinderivatives: 2.5 μmol/L paclitaxel: 2.5 μmol/L	HepG2 SMMC-7721	Induced apoptosis by increasing microtubule dynamics and the sensitivity of paclitaxel	(148)
Curcumin/ Paclitaxel	curcumin: 5-20 μmol/L paclitaxel: 1 μmol/L	Hep3B HepG2	Induced apoptosis, and reversed the drug resistance of paclitaxel through inhibiting NF-κB pathway stimulated Lin28B expression	(149)
Folate/ Paclitaxel	FA-PEG-MSNs-PTX: 100 μg/mL	SMMC-7721	Had a good targeting effect on SMMC-7721 cells and enhanced the anti-tumor effect of paclitaxel	(150)
Esculetin/ Paclitaxe	esculetin: 50-100 μmol/L paclitaxe: 0.5 μmol/L	HepG2	Induced apoptosis through up-regulating Bax/Bcl, inducing the activation of caspase-8 and caspase-3, as well as ERK signal pathway	(151)
Resveratrol/ Paclitaxel	resveratrol: 10 μg/mL paclitaxel: 5, 10 μg/mL	HepG2	Up-regulated P53, Bax and other proteins, and reversed drug resistance of paclitaxel	(152)
Emodin/ Curcumin	emodin: 10 mg/L curcumin: 10 mg/L	BEL-7402	Enhanced sensitivity and reduce the dosage of emodin and induce apoptosis	(153)
Curcumin/ Paclitaxel	curcumin: 5-20 µmol/L paclitaxel: 0.05-0.2 µmol/L	Hep3B	Decreased paclitaxel-induced NF-κB activation, mediated Lin28 level, thereby enhancing sensitivity of liver cancer cells to paclitaxel	(154)

## The synergistic effect of plant-derived natural products in combination with chemotherapy

Clinical chemotherapy drugs mainly include cisplatin which is platinum compounds, and doxorubicin which is antibiotics, and hormones. Cisplatin and adriamycin mainly affect cell division and induce apoptosis by interfering with DNA replication (156). However, chemotherapeutic drugs significantly reduce the immune function of the body, and induce multiple organ damage (157, 158). Radiation and chemotherapeutic agents as the backbone for cancer are not very effective and toxic not only to tumor cells but also to normal cells, and are not affordable for most. Therefore, plant-derived natural products in combination with chemotherapeutic drugs are generally free of deleterious side effects and usually inexpensive, which have played a significant role in the development of anticancer drugs over the years. It remains to mention that extraction from natural plants is also contributed to the synergistic effects with other anticancer drugs such as cisplatin, doxorubicin, and hormones. Plant-derived natural products in combination with chemotherapeutic agents act as an anticancer agent by inhibition of tumor cell angiogenesis, invasion and migration, induction of autophagy.

### Suppress liver cancer cells angiogenesis

Liver cancer is a kind of tumor rich in blood vessels. And blood vessels providing essential nutrients and oxygen for the occurrence and development of liver cancer cells, and are closely related to the growth and metastasis of liver cancer cells (159). Vascular endothelial growth factor (VEGF) is the most important type of angiogenesis regulatory factor, which promotes the division and proliferation of vascular endothelial cells through paracrine and promoting formation of blood vessels (160). Angiopoietin (Ang)/Tie2 pathway regulates vascular and lymphatic development, vascular permeability, angiogenesis remodeling, and tumor vascularization (161, 162). Tumor cells act by up-regulating VEGF through inducing miR-21 targeting AKT and ERK signaling pathways, thereby promoting tumor angiogenesis (163). The mTOR pathway can also promote tumor angiogenesis through inducing the transformation of tumor-associated macrophages (TAM) into M2-type. Therefore, it can reduce proliferation and metastasis of liver cancer cells through preventing the formation of new blood vessels (164, 165).

Ao et al. found that the main component of kanglite injections is the extract from coix seed in traditional Chinese medicine, this active compound in combination with thalidomide can significantly inhibit human liver cancer blood vessels through inhibiting VEGF and B-FGF, thereby inducing angiogenesis. At the same time, it can regulate cellular immunity of the body, which has synergistic effect with chemotherapy (166). Compared with cisplatin group, the alcohol extracts of *Liuwei rehmannium* (mainly composed of polysaccharide, paeonol, flavonoids, trace elements, etc.) in combination with cisplatin, can significantly induce apoptosis of BEL-7402 cells, and inhibit angiogenesis by down-regulating the expression of VEGF and ANG-2 proteins, up regulating the expression of TSP and TIMP-2 (167). Lin et al. reported that compared to model group, a combination of astragalus polysaccharide with cisplatin and adriamycin could significantly reduce the expressions of Ki-67, HIF-1α and VEGF, and had a significant synergistic and toxic effect on inhibition of tumor growth (168). Zang et al. confirmed that radix astragali or curcuma in combination with cisplatin acted by inhibiting angiogenesis and metastasis of liver cancer through down-regulating the expression of CD147 and iNOS (169). Luo et al. showed that a combination of cisplatin with astragaloside IV or curcumin which is flavonoid compound have a significant inhibition the formation of new blood vessels in liver cancer by down-regulating the expression of HIF-1α and VEGF (170). Chen et al. researched that combining epigallocatechin gallate (EGCG) derivative Y6 with dunorubicin which is anthracycline antitumor drug, acts by inhibiting of angiogenesis and toxic effects *in vivo* through inhibiting of MAPK/ERK and PI3K/AKT signaling pathways to down-regulate the expression of HIF-1α, Carbonyl Reductase1 (CBR1) and VEGF (171). Zhao et al. developed doxorubicin (DOX) and curcumin (Cur) co-delivery lipid nanoparticles (DOX/Cur-NPs) and examined its inhibitory effect on diethylnitrosamine (DEN)-induced liver cancer in mice. The mRNA levels of MDR1, Bcl-2 and HIF-1α, and protein levels of P-gP, Bcl-2 and HIF-1α were decreased in DOX/Cur-NPs than those in DOX-NPs, indicating that Cur might reverse multidrug resistance (MDR) through these pathways (172).

### Inhibit invasion and migration of liver cancer cells

A critical event during tumorigenesis is the transformation from a static primary tumor to an aggressive and invasive oner. During the processes, tumor cells show an increased capacity to migrate, and multitudinous intracellular molecules participate in migration. Metastasis is significantly pathological characterized by malignant tumors, while the invasion and migration of liver cancer cells lead to poor prognosis of liver cancer patients, prone to recurrence and short median survival (173). The invasion and migration of tumor cells is a complex process controlled by multiple factors (174). It mainly included EMT, inhibition of matrix metalloproteinases (MMPs) hydrolysis and cell adhesion to basement membrane and extracellular matrix (175). And it is affected by the regulation of tumor metastasis genes and tumor metastasis suppressor genes (176). Combining natural products with chemotherapy drugs can prevent and cure cancer metastasis safely and effectively.

Shan et al. confirmed that mannose and cisplatin had a synergistic effect, which inhibited proliferation and migration of HepG2 cells, furthermore, it can change morphology with separation, shedding and aggregation (177). A combination of emodin with cisplatin can up-regulate the expression of E-cadherin, acts by inhibiting proliferation, invasion and migration of HepG2 cells through inhibiting EMT (178). Yang et al. showed that kaempferol and doxorubicin synergistically inhibited the proliferation, migration and invasion of liver cancer cells by inhibiting PI3K/mTOR/MMP protein pathway in a dose-and time-dependent manner (179). The sinosinine thiocyanate extracted from the herbaceous plants of The Cross family, could target the multidrug-resistant related proteins ABCB1 and ABCG2, enhance the sensitivity of HepG2 cells to gemcitabine, and inhibit the invasion and migration (180). Wang et al. conducted an investigation about the potential anti-tumor mechanism of alkaloid caffeine in combination with 5-fluorouracil. It can exhibit a synergistic anti-liver cancer effect by increasing the expression of cleaved PARP, p-JNK, p-p38, and decreasing the expression of Bcl-2, Bcl-xL, CDK2/4/6, p-ERK, p38, *via* inducing generation of ROS and influencing MAPK signaling pathway, thereby inhibiting the proliferation and metastasis and inducing apoptosis of SMMC-7721 and Hep3B cells (181). Data from a study *in vitro* revealed that combined metformin (10 mmol/L) and curcumin (5 and 10 µmol/L) could induce apoptosis and inhibit metastasis in HepG2 and PLC/PRF/5 cells. The anticancer effects could be attributed to inhibition of VEGF, MMP2/9, and VEGFR-2 protein expression (182).

### Induce liver cancer cells autophagy

Autophagy is a genetically controlled and evolutionarily conserved form of cell death, which is also related to occurrence and development of tumor, thereby has becoming a new tumor therapy method. Several cellular classical signaling pathways of natural plants target intracellular autophagy including Beclin-1, phosphoinositide 3-kinase/Akt/mechanistic target of rapamycin pathway (PI3K/AKT/mTOR) and p53, have been known to control cell proliferation and apoptosis (183, 184). Beclin-1 and LC3 are two key markers of autophagy. Beclin-1 can enhance autophagy and induce apoptosis of tumor cells (185). p53 can induce autophagy by activating cathepsin D (CTSD), and further enhance the p53-mediated tumor suppression effect (186). In addition, p53 can activate the expression of transglutaminase 2 (TGM 2) to promote autophagy and tumor suppression functions, and TGM 2 contributes to the development of p53-induced autophagy program and the function of tumor suppressor (187). Moreover, autophagy is also regulated by p53 in the elimination of aberrant mitochondria. In radiation-resistant cancer cells, p53 induces mitophagy by increasing the levels of BCL 2 interacting protein 3 (BNIP 3) to remove abnormal mitochondria to maintain mitochondrial oxidative phosphorylation (OXPHOS) and inhibit the glycolytic pathway (188). Importantly, PI3K-AkT-mTOR signaling pathway is a well-characterized anti-apoptotic cascade in human cancers, since its activation leads to cell proliferation and cell survival. The inducing cell survival of this pathway was mediated by activating anti-apoptotic factors and inhibiting pro-apoptotic factors. AKT can induce anti-apoptotic effect through phosphorylating target proteins by a variety of pathways, such as prevent the apoptosis cascade through inhibiting caspase-9 activity (189). Natural products act by inducing autophagy and apoptosis of cells through down-regulating the expression of mTOR, AKT, PI3K and other proteins (190).

Hu et al. suggested that a combination of matrine with cisplatin promoted apoptosis through activating the caspase apoptosis pathway and inhibiting expression of survivin-related caspase-9 protein (191). A combination of tannic acid with cisplatin acts by inducing apoptosis through activating caspase-3 signaling cascade (192). Xu et al. revealed that saikosaponin D in combination with doxorubibin significantly induced autophagy and apoptosis of HepG2 cells. It suppressed cell proliferation and invasion through up-regulating of ROS, Oatp1b1 as well as TGF-β1 (193). Wang et al. showed that ginsenosides in combination with anti-cancer drugs regorafenib acted by inhibiting growth of HepG2 cells through regulating the expression of Survivin and Caspase-3 genes (194). Ginkgol C17:1 inhibited cisplatin-induced autophagy *via* AMP-activated protein kinase/ULK1signaling and increased cisplatin-induced apoptosis in HepG2 cells *via* the PI3K/AKT/mTOR signaling pathway (195). Astragaloside IV effectively protected against cisplatin-induced injury by inducing autophagy and limiting the expression of NLRP3 (196). Aloin in combination with metformin synergistically inhibited HepG2 and Bel-7402 cell proliferation and invasion and induced apoptosis and autophagy through activating PI3K/AKT/mTOR pathway *in vitro*. In addition, it has a synergistic effect by inhibiting growth and promoting apoptosis and autophagy in HepG2 xenograft mice model (197). Sinapic acid combined with cisplatin inhibits proliferation and migration of HepG2 and SMMC-7221 cells. At the same time, this combination therapy induces autophagy by upregulating the protein expressions of LC3II, Beclin-1 and Atg5, and downregulating the expression of p62 (198). The combination of 6-shogaol and 5-FU inhibit AKT/mTOR/MRP1 signaling pathway and induce liver cancer cell apoptosis by decreasing the expression of AKT, mTOR, MRP1 and cyclin-related proteins (199).

With the development of nanotechnology, nano-drug delivery system can improve drug solubility improve drug stability, increase drug targeting, reduce drug toxicity and side effects. Ceria Nanoparticles can kill cancer cells through induce cancer cells to produce ROS, reduce the expression level of various antioxidant enzymes, cause mitochondrial damage and apoptosis (200). Xu et al. synthesized a composite material DCQ which dextran-coated cerium oxide nanoparticlesloaded quercetin, and discovered that the composite material was more toxic to HepG2 cells, and had no obvious toxic effect on normal cells, human umbilical vein endothelial cells (HUVEC). The composite material can induce the production of ROS, inhibit the fusion of autophagosomes and lysosomes in HepG2 cells and cause the accumulation of autophagosomes, block autophagy and promote apoptosis of HepG2 cells (201). Dong et al. synthesized a nano-layered double hydroxide (NLDH) co-loaded fluorouracil (Fu) and curcumin (Cur) mixed dosage form (LDH-Fu-Cur, LFC). Compared with pure fluorouracil, fluorouracil plus curcumin, and LDH-Fu, LDH-Fu-Cur more efficiently inhibited the growth and promoted the apoptosis of 7721, LM3 and Hep G2 cell lines. It also could significantly down-regulate the Bcl-2 gene expression of 7721 cells and induce cell apoptosis by activating caspase 3 and caspase 9 (202). Moreover, han explored a unique amphiphilic PCL-AuNC/Fe (OH)3-PAA Janus nanoparticle (JNP) to simultaneously preserve the hydrophilic drug (doxorubicin) and hydrophobic drug (docetaxel) in their distinct domains. It realized the optional sequential drug release by a single inorganic JNP for the first time, and the simultaneous release of the two drugs can improve efficacy and reduce toxic side effects. Furthermore, the mice treated with dual drug loaded PCL-AuNC/Fe (OH)3-PAA JNPs under near infrared (NIR) laser irradiation showed better tumor inhibition than solo drug, cocktail and dual drug treated groups, induced aopotosis and blocked the cell cycle of HepG2, and inhibited the growth of tumor *in vivo*, indicated the effectivity and significance of combined cancer therapy (203). To improve the utilization of curcumin (CUR) and 5-fluorouracil (5-FU) chemotherapeutic drugs in the treatment of liver tumors, increase the accumulation of drugs in tumor sites and reduce the side effects of drugs on the systemic system, Ni Wenfeng constructed a novel targeting nanocarrier to transport the drug to the liver tumor site. In their study, an amphiphilic triblock PEG-PLGA-PEG copolymer was coupled with two targeting ligands (BIO and LAC) to prepare dual-targeted nanoparticles (BLPPNPs), which encapsulated curcumin (CUR) and 5-FU. And the mechanism of synergistic anti-hepatocarcinogenesis of the two drugs may be that CUR down-regulates the expression of DPYP protein by up-regulating the expression of p53 protein, increasing the cytotoxicity of 5-FU and enhancing the anti-tumor effect. At the same time, CUR may decrease the expression of Bcl-2 protein, increase the release of cyt c, and promote the apoptosis of hepatoma cells (204). Zhang successfully prepared a kind of multifunctional nanoparticles: Janus-magnetic mesoporous silica nanoparticles (Fe_3_O_4_-MSNs), with the surface modified by hyaluronic acid (HA) and nano fluorescent probe quantum dots (QD), and chemotherapy drug doxorubicin and berberine (ber), Fe_3_O_4_-MSN/Dox+Ber@HA-QD. The *in vitro* experiments showed that the nanoparticles could selectively enter HepG2 cells through the CD44 receptor mediated endocytosis pathway and release dox and rer rapidly, which had better cytotoxic effects at low concentrations. The *in vivo* results also suggested that the nanoparticles have a significantly lower recurrence of the hepatocellular carcinoma induced by dox and significantly reduced the toxic and side effect of doxorubicin while having a better antitumor effect than other groups. In addition, the nanoparticles can also achieve MRI and fluorescence imaging of the tumor site, providing the possibility of drug delivery and therapeutic effects (205). **Table 2** summarizes their effects and mechanisms on liver cancer.

**Table 2:** Effect and mechanism of plant-derived natural products combination Chemotherapy drugs.

Combination Therapy	Concentration	Cell Line/Model	Mechanism	Reference
Kanglite injection/ Thalidomide	kanglite injections: 200 mL/d thalidomide: 100 mg/d	76 patients of primary liver cancer	Inhibited blood vessels through inhibiting VEGF and B-FGF induced angiogenesis, and regulate cellular immunity of the body	(166)
Liuwei Dihuang/ Cisplatin	liuwei dihuang: 100 mg/mL cisplatin: 1 mg/mL	BEL-7402	Downregulated the expressions of VEGF and Ang-2, upregulated the expression of TSP and TIMP-2	(167)
Astragalus polysaccharide/ Cisplatin/ Doxorubicin	astragalus polysaccharide: 50-200 mg/kg cisplatin: 2 mg/kg doxorubicin: 6 mg/kg	HepG2 tumor-bearing mice	Reduced the expressions of Ki-67, HIF-1α and VEGF	(168)
Radix astragali/ Curcuma/ Cisplatin	radix astragali: 3-12 g/kg/d curcuma: 3-12 g/kg/d cisplatin: 2 mg/kg	HepG2 tumor-bearing mice	Down-regulated the expression of CD147 and In-OS	(169)
Astragaloside IV/ Curcumin/ Cisplatin	astragaloside IV: 12 g/kg/d curcumin: 10 g/kg cisplatin: 2 mg/kg	HepG2 tumor-bearing mice	Inhibited the formation of new blood vessels in liver cancer by down-regulating the expression of HIF-1α and VEGF	(170)
Epigallocatechin gallate/Derivative Y6/ Daunrrubicin	EGCG: 40 mg/kg Y6: 27.5-110 mg/kg daunrrubicin:2 mg/kg	HepG2 tumor-bearing mice	Inhibited MAPK/ERK and PI3K/AKT signaling pathways and down-regulated the expression of HIF-1 α, CBR1 and VEGF	(171)
Curcumin/ Doxorubicin	DOX/Cur-NPs: 2 mg/kg	Liver cancer mice	Decreased the mRNA levels of MDR1, Bcl-2 and HIF-1α, and protein levels of P-gP, Bcl-2 and HIF-1α	(172)
Mannose/ Cisplatin	mannose: 25 mmol/L cisplatin: 10 µmol/L	HepG2	Inhibited proliferation and migration and changed morphology with separation, shedding and aggregation	(177)
Emodin/ Cisplatin	emodin: 6.25-50 µg/mL cisplatin: 2.5 µg/mL	HepG2	Up-regulated the expression of e-cadherin, and inhibited EMT	(178)
Kaempferol/ Doxorubicin	kaempferol: 0-40 mmol/L doxorubicin: 300-1200 nmol/L	Huh-7, Huh-1, HepG2 HepG2.2.15 SK-Hep-1 PLC/PRF/5 HLE, HLF, Hep3B	Inhibited PI3K/mTOR/MMP protein pathway in a dose-and time-dependent manner153	(179)
Sinosinine thiocyanate/ Gemcitabine	sinosinine thiocyanate: 50 µmol/L gemcitabine: 0-20 µmol/L	HepG2	Inhibited the expression of multidrug-resistant related proteins ABCB1 and ABCG2,155 enhance the sensitivity to gemcitabine	(180)
Caffeine/ 5-fluorouracil	caffeine: 0.5-1 mmol/L 5-fluorouracil: 0-50 µmol/L	SMMC-7221 HepG3B	Increased the e156xpression of cleaved PARP, p-JNK, p-p38, and decreased the expression of Bcl-2, Bcl-xL, CDK2/4/6, p-ERK, p38, *via* inducing generation of ROS and influencing MAPK signaling pathway,	(181)
Curcumin/ Metformin	curcumin: 5-10 µmol/L metformin: 10 mmol/L	HepG2 PLC/PRF/5	Inhibted the expression of VEGF, MMP2/9, and VEGFR-2	(182)
Matrine/ Cisplatin	matrine: 100 mg/kg cisplatin: 2 mg/kg	HepG2 nude mice	Activated the caspase apoptosis pathway and inhibiting expression of Survivin-related caspase-9 protein	(188)
Tannic acid/ Cisplatin	tannic acid: 90-540 µmol/L cisplatin: 0.6-3.6 µg/L	HepG2	Induced apoptosis through activating caspase-3 signaling cascade	(192)
Saikosaponin D/ Doxorubibin	saikosaponin D: 2-16 µg/mL doxorubibin: 0.5-16 µg/mL	H22 mice model	Suppressed cell proliferation and invasion through up-regulating of ROS, Oatp1b1 as well as TGF-β1	(193)
Ginsenoside/ Regorafenib	ginsenoside: 10 mg/L regorafenib: 1 mg/L	HepG2	Regulated the expression of Survivin and Caspase-3 genes	(194)
Ginkgol C17:1/ Cisplatin	ginkgol C17:1: 0-80 µg/mL cisplatin: 2 µg/mL	HepG2	Induced AMP-activated protein kinase/ULK1signaling and the phosphoinositide 3-kinase/Akt/mechanistic target of rapamycin pathway	(195)
Astragaloside IV/ Cisplatin	astragaloside IV:40-80 mg/kg cisplatin:15 mg/kg	Liver cancer SD rats	Induced autophagy and limiting the expression of NLRP3	(196)
Aloin/ Metformin	aloin: 50 μmol/L metformin: 400 μmol/L	HepG2 Bel-7402	Activated PI3K/AKT/mTOR pathway	(197)
Sinapic acid/ Cisplatin	sinapic acid: 0-2,000 μmol/L cisplatin: 5 μmol/L	HepG2 SMMC-7721	Upregulated the protein expressions of LC3II, Beclin-1 and Atg5, and downregulated the expression of P62	(198)
6-shogaol/ 5-fluorouracil	6-Shogaol: 10 μmol/L 5-fluorouracil: 10 μmol/L	HepG2 Li-7	Inhibited AKT/mTOR/MRP1 signaling pathway and decreased the expression of AKT, mTOR, MRP1 and cyclin-related proteins	(199)
Quercetin/ Ceria nanoparticles	DCQ: 33-165 μmol/L	HepG2	Induced the production of ROS, inhibited the fusion of autophagosomes and lysosomes in HepG2 cells and caused the accumulation of autophagosomes, blocked autophagy and promoted apoptosis of HepG2 cells	(201)
Curcumin/ Fluorouracil	LFC: 15-35 µg/mL	LM3 Hep G2 7721	Down-regulated the Bcl-2 gene expression of 7721 cells and induced cell apoptosis by activating caspase 3 and caspase 9	(202)
Docetaxel/ Doxorubicin	PCL-AuNC/Fe(OH)_3_-PAA JNPs: 1.56-100 µg/mL	HepG2/H22 BALB/c tumor-bearing mice	Induced apoptosis and blocked the cell cycle of HepG2, and inhibited the growth of tumor *in vivo*	(203)
Curcumin/ 5-fluorouracil	combined feeding ratio: 1:2.5 (5-FU: CUR)	HepG2 HL7702 HepG2 tumor-bearing nude mice	Down-regulated the expression of DPYP protein by up-regulating the expression of p53 protein, increasing the cytotoxicity of 5-FU and enhancing the anti-tumor effect, decreased the expression of Bcl-2 protein, increased the release of cyt c, and promoted the apoptosis of hepatoma cells	(204)
Berberine/ Doxorubicin	Fe_3_O_4_-MSN/Dox+Ber@HA-QD: 4 mg/kg	HepG2 H22 ICR tumor-bearing mice	Reduced the toxic and side effect of doxorubicin while having a better antitumor effect than other groups	(205)

## Anti-liver cancer mechanism of plant-derived natural products in combination with immunotherapy

The immune system of the body can recognize and eliminate foreign invading antigens, mutated cells and senescent cells of the body, and maintain the stability of the internal environment. However, tumor cells have the ability to escape immune surveillance, immune identification, and eradication, and immune system dysfunction lead to the development of malignant cells with clinical manifestationsb (206). According to the immune escape mechanism of tumor cells, who established a variety of immunotherapies of cancer (207). This kind of breakthrough therapy is defined that regulates immunological response through activating the organism’s immune defense system or action of biological compounds to suppress and prevent tumor growth. At present, the commonly immunotherapy methods include immune checkpoint blockade therapy (208), antibody therapy (209), tumor vaccine therapy (210). Cancer immunotherapy has become an irreversible trend herald in the field of cancer therapy and is regarded as the fourth type of anti-tumor treatment after surgery, radiotherapy, and chemotherapy due to the obvious efficacy and low side effects. Although immunotherapy has made significant progress in the treatment of liver cancer, it needed to be further improved the clinical efficacy, find more specific immune targets, while avoid unnecessary targeting and off-target toxicity (211). Therefore, a combination of natural products and tumor immunotherapy can learn from each other and coordinate with each other to improve the effective rate of treatment.

### Regulate immune function

It has been known that immune function of body is closely related to the occurrence and development of tumors. As host immune function is low or suppressed, the incidence of tumor will be increased. And in the progressive growth of tumor, the immune function will also be inhibited by the tumor. These two factors are mutual causation and the fluctuation directly affects the occurrence and development of tumor. Tumor microenvironment is an extremely complex system that involves several kinds of multifunctional immunizing cells and molecules, as well as tumor cells. It is a dynamic system composed of cancer cells, cytokines, extracellular matrix and immune cell subsets (212). Formation of Individual tumor is likely to develop because the tumor-associated microenvironment promotes tumor cell growth and protects tumor cells from elimination of systemic effector cells (213). Natural products in combination with immunotherapy act by inducing antitumor effects through restoring immune recognition and immune elimination by regulating the immune function.

Hu et al. revealed that *Lycium barbarum* polysaccharide in combination with cytokine CXCL10 acts by improving immune function against liver cancer. It also promotes secretion of Th1 cytokines and restores balance of Thl/Th2, improving number and function of dendritic cells (DC) and intervening immune suppression state of tumor-bearing bodies (214). Chen et al. demonstrated that a combination of anti-PD-L1 antibody and high concentration of vitamin C acts by promoting the expression of PD-L1 *in vivo* and *in vitro* and enhance the efficacy of anti- PD-L1 antibody. It can promote liver tumor blood vessels normalization and eventually increase T cell infiltration through activating cyclic GMP-AMP synthase (cGAS) and promoting the secretion of its product cGAMP, and then activating the STING pathway of vascular endothelial cells (215). Song et al. proved that a combination of 10-hydroxycamptotenin and DC hepa-6 fusion vaccine acts by inducing improving immune function and significantly inducing CTL cytotoxicity, and resisted against the rechallenge of Hepal-6 cells (216). Yang et al. reported that Chinese herb medicine combine with targeted therapy of TCM immune for patients with end-stage liver cancer, after treatment, improved symptoms of abdominal distension and jaundice, nutritional status and quality, alleviated the pain, and prolonged overall survival time (217). Yu et al. suggested that sorafenib combined with Poria Lingsini decoction can significantly improve immune function and the survival outcome of patients with advanced primary liver cancer (218).

### Induce liver cancer cell apoptosis

Endoplasmic reticulum (ER) stress results from the accumulation of unfolded or misfolded proteins in the ER, is involved in regulating apoptotic process in tumor cells (219). It will trigger endogenous and exogenous apoptotic signals as ER dysfunction persists in eukaryotic cells, in addition, the heavy and continuing endoplasmic reticulum stress can lead to cell dysfunction and even cell death. When endoplasmic reticulum stress (ERS) occurs in cells, the expression of anti-apoptotic molecule BiP (immunoglobulin binding protein) and pro-apoptotic molecules, such as CHOP and caspase-12, is regulated to determine whether the cells will undergo apoptosis or survive to adapt to the environment (220). Plant-derived natural products in combination with immunotherapy can induce apoptosis of tumor cells.

Jin et al. prepared paclitaxel-loaded nanoparticles decorated with anti-CD133 antibody, which could effectively target liver cancer stem cells and induce apoptosis of liver cancer cells (221). Wang et al. demonstrated that paclitaxel in combination with anti-human stathmin1 antibody inhibits proliferation and induces apoptosis of liver cancer cells (222). Lin et al. found that the combination of taxol and immunosuppressant cyclosporin A increases the expression of caspase-9 and caspase-3, induces apoptosis of liver cancer cells through PI3K-mTOR pathway, and effectively reverses drug resistance of paclitaxel (223). Huang et al. confirmed that a combination of gambogic acid and proteasome inhibitors induces liver cancer cell apoptosis and inhibits growth through inducing cytotoxicity and enhancing proteasome inhibition and ER stress (224). A combination of n-glycosylation inhibitor tunicamycin and camptothecin acts by inducing resistance through up-regulating GRP78 and blocking G1 phase cell cycle (225). Nicotinamide and STAT3 inhibitor significantly induced apoptosis and inhibited proliferation by reducing phosphorylation of STAT3 Y705 in HepG2 cells, down-regulating the expression of SNAIL1, VEGFA and ZEB1, EMT-related gene and glucose metabolism. Moreover, the synergistic effect is more significant (226).

Photodynamic therapy (PDT), with its unique advantages of minimally invasive, good selectivity, high safety and unique efficacy, has been used as a new therapeutic method for liver tumor treatment. As a natural product, hypocrellin B (HB) can be used as a photosensitive agent for PDT treatment of many diseases, especially for tumor treatment. In order to improve poor water solubility, fast metabolism *in vivo* and no specific tissue distribution of HB, zhang collected liver cell membrane (CCCM) and modified tumor-targeting ligand (transferrin, TF) into homologous liver cancer cell membrane (TF-CCCM), and prepared ethylene nanoparticles (HB NPs) by double emulsion method. Then TF-CCCM-HB NPs was prepared by extrusion method by mixing the two in a certain proportion. TF-CCCM-HB NPs has strong targeting ability and phagocytosis in homologous liver cells, and suppress the proliferation of tumor cells in a dose-dependent manner. Moreover, it has a synergistic effect with PDT, which can promote the production of ROS and reduce mitochondrial membrane potential. At the same time, TF-CCCM-HB NPs combined with PDT can inhibit the growth of tumor in mice model with liver cancer, prolong the survival time of tumor-bearing mice, and inhibit tumor metastasis and recurrence, which has good biosafety (227). Bai prepared a form drug/gene co-loaded nanoparticles (sCDP/DOX/miR-122), which a nanosystem based on β-cyclodextrin-cored star polymer (sCDP) and co-deliver antitumor drug doxorubicin (DOX) and miRNA-122. s CDP/DOX/miR-122 nanoparticles can remarkably inhibit HepG2 cell proliferation, promote cell apoptosis and increase the expression of p53 and cleaved caspase 3; it also can downregulate the expression of Bcl-w and CCNG1 leading to irreversible cell apoptosis, decrease the expression of MDR1, MRP and P-gp, and improve the sensitivity to DOX of HepG2 cells, thus realizing the synergistic antitumor effect *in vitro*. sCDP/DOX/miR-122 nanoparticles can slow down the weight loss of nude mice bearing tumor and significantly inhibit tumor growing, thus realizing a synergistic anti-tumor effect *in vivo* (228). Yuan synthesis iron oxide nanoparticles loaded polydopamine (PDA) and doxorubicin (Dox). Rats were treated with hepatic artery *via* interventional methods, and it was verified that Fe_2_O_3_-PDA-Dox nanoparticles can simultaneously have the ecceft of Transcatheter arterial chemoembolization (TACE) and photothermal ablation (PTA). It also confirmed that combretastatin A-4 phosphate disodium (CA4P) can increase the uptake of Fe_2_O_3_-PDA-Dox nanoparticles by rat hepatocellular carcinoma, which can enhance the combined treatment of transcatheter arterial chemoembolization and photothermal ablation without additional increase in liver and kidney toxicity (229).

### Reduce liver cancer cells metastasis

Plant-derived natural products in combination with immunotherapy act by inhibiting tumor cell proliferation and metastasis through inhibiting angiogenesis and arresting cell cycle. Lai Chunhui prepared nanoparticles which mannose (M)/CpG oligodeoxynucleotide (CpG-ODN)-conjugated liposomes lipo loaded with tumor-associated antigens. It specifically induced the activation and maturation of DCs *in vivo* and the activated DCs stimulated effector cells to kill tumor cells in mice, thereby achieving the effect of anti-tumor immunotherapy (230). Zhong et al. revealed that tubacin, an inhibitor of histone deacetylase 6 (HDAC6), in combination with docetaxel, could arrest the cell cycle, inhibit metastasis and proliferation, as well as induce apoptosis of liver cancer cells (231). Shen et al. found that evocorine in combination with autophagy inhibitor chloroquine (CQ) acts by inhibiting angiogenesis of HepG2 cells through inducing autophagy. It can decrease the expression of VEGFA and inhibit invasion (232). Lai et al. demonstrated that evodiamine or paclitaxel in combination with CDK1 inhibitors acts by inducing the apoptosis of hepatocarcinoma cells through arresting G2/M phase cell cycle. Evodiamine in combination with R03306 have a synergistic effect on upregulating the expression of cyclin E and decreasing the expression of cyclin B1. Moreover, paclitaxel combined with R03306 can decrease the expression of Bcl-2 and cyclin B1, increase the expression of Bax, thereby inducing apoptosis in HepG2 cells (233). Compound kushen injection significantly enhanced the anticancer activity of sorafenib at a subclinical dose without obvious side effects, and prevented the postsurgical recurrence and rechallenged tumor growth. It activated proinflammatory responses and relieved immunosuppression of tumor-associated macrophages in the liver cancer cell microenvironment by triggering tumor necrosis factor receptor superfamily member 1 (TNFR1)-mediated NF-κB and p38 MAPK signaling cascades, which subsequently resulted in apoptosis of liver cancer cells (234). **Table 3** summarizes their effects and mechanisms on liver cancer.

**Table 3:** Effect and mechanism of plant-derived natural products in combination with immunotherapy.

Combination Therapy	Concentration	Cell Line/Model	Mechanism	Reference
Lycium barbarum polysaccharide/ CXCL10	lycium barbarum polysaccharide: 100 mg/kg CXCL10: 15 µg/kg	H22 mice model	Improved immune function and promoted secretion of Th1 cytokines and restore balance of Thl/Th2, improved number and function of DC cells and intervened immune suppression state of tumor-bearing bodies	(214)
Vitamin C/ Anti-PD-L1 antibody	vitamin C: 4 g/kg Anti-PD-L1 antibody: 75 µg/3day	Hepa1-6 cancer mice	Activated cyclic GMP-AMP synthase (cGAS) and promoted the secretion of its product cGAMP, and then activated the STING pathway of vascular endothelial cells	(215)
10-hydroxycamptothecinc/DC hepa1-6fusion vaccine	10-hydroxycamptothecinc: 50-100 µg/mL	Hepa1-6 cancer mice	Induced improving immune function and inducing significantly CTL cytotoxicity	(216)
Chinese herb medicine/ Camrelizumab/ Lenvatinib	camrelizumab: 200 mg/3weeks lenvatinib: 8 mg/day	Advanced liver cancer patient	Improved symptoms of abdominal distension and jaundice, nutritional status and quality, alleviated the pain, and prolonged overall survival time	(217)
Fuling sini decoction/Sorafenib	sorafenib: 800 mg/day	Liver cancer patient	Improved the survival outcome of patients with advanced primary liver cancer, improve immune function	(218)
Paclitaxel/ Anti-CD133 antibody	nanoparticles: 7.51-56.92 ng/mL	Huh-7 HepG2	Targeted liver cancer stem cells and induced apoptosis	(221)
Paclitaxel/ Anti-human stathmin1antibody	paclitaxel: 0.1-1.6 µg/mL anti-human stathmin l antibody: 10-160 µg/mL	HepG2	Inhibited proliferation and induced apoptosis	(222)
Taxol/ Cyclosporin A	taxol: 0.1 µmol/L cyclosporin A: 0-10 µmol/L	Hep3B HepG2 HA22T VGH Hepa 1-6	Increased the expression of caspase-9 and caspase-3, induced apoptosis of liver cancer cells through PI3K-mTOR pathway, and effectively reversed drug resistance of paclitaxel	(223)
Gambogic acid/ Bortezomib	gambogic acid: 0.4-0.6 µmol/L bortezomib: 40-60 nmol/L	HepG2 H22	Induced cytotoxicity and enhanced proteasome inhibition and ER stress	(224)
Camptothecin/ Etoposide/ Tunicamycin	camptothecin:3 μmol/L etoposide: 5 μmol/L tunicamycin: 1 μg/mL	Hep3B	Induced resistance through up-regulating GRP78 and block G1 phase cell cycle	(225)
Nicotinamide/ STAT3 inhibitor	nicotinamide: 5 mmol/L S3I-201:100 µmol/L stattic: 1.5 µmol/L	HepG2	Reduced phosphorylation of STAT3 Y705, down-regulated the expression of SNAIL1, VEGFA and ZEB1, and decreased epithelial-mesenchymal transition and glucose metabolism	(226)
Hypocrellin B/ Transferrin/ Membrane	TF-CCCM-HB-NPs: 0-100 µg/mL	HepG2 LO2 HepG2 tumor-bearing mice model	Promoted the production of ROS and reduced MMP, inhibited the growth of tumor in mice model with liver cancer, prolonged the survival time of tumor-bearing mice	(227)
Doxorubicin/ miRNA-122	sCDP/DOX/miR-122: 2.1 µg/mL	Hep G2 Liver nude mice bearing tumor	Increased the expression of p53 and cleaved caspase-3, downregulated the expression of Bcl-w and CCNG1 leading to irreversible cell apoptosis, decreased the expression of MDR1, MRP and P-gp, and improved the sensitivity to DOX of HepG2 cells	(228)
Doxorubicin/ Combretastatin A-4 phosphate disodium	Fe_2_O_3_-PDA-Dox: 0.4 mg/mL CA4P: 0.1 mg/mL	Liver tumor rats model	Enhanced the combined treatment of transcatheter arterial chemoembolization and photothermal ablation without additional increase in liver and kidney toxicity	(229)
Mannose/ Tumor-assoclated antigens	M/CpG-ODN-H22-Lipo: 100 µL/mice	H22 bearing mice model	Induced the activation and maturation of DCs *in vivo* and the activated DCs stimulated effector cells to kill tumor cells in mice	(230)
Docetaxel/ Tubacin	docetaxel: 0-32 µmol/L tubacin: 10 µmol/L	SNU449 SNU387	Arrested the cell cycle, inhibit metastasis and proliferation, as well as induce apoptosis of liver cancer cells	(231)
Evodiamine/ Chloroquine	evodiamine: 10 μmol/L chloroquine: 25 μmol/L	HepG2	Inhibited angiogenesis through inducing autophagy, and decreased the expression of VEGFA and inhibited invasion	(232)
Evodiamine/ Paclitaxel/ CDK1 inhibitors	evodiamine:1-4 μmol/L paclitaxel: 0.2 μmol/L R0306: 2 μmol/L	HepG2	Decreased the expression of Bcl-2 and cyclin B1, increased the expression of Bax and cyclin E	(233)
Compound kushen injection/ Sorafenib	compound kushen injection: 0.43-1.32 mg/mL sorafenib: 10, 30 mg/kg	Hepa1-6 tumor nude mice	Activated proinflammatory responses and relieved immunosuppression of tumor-associated macrophages in the liver cancer cell microenvironment by triggering tumor necrosis factor receptor superfamily member 1 (TNFR1)-mediated NF-κB and p38 MAPK signaling cascades	(234)

## Conclusion

**Figure 2:**
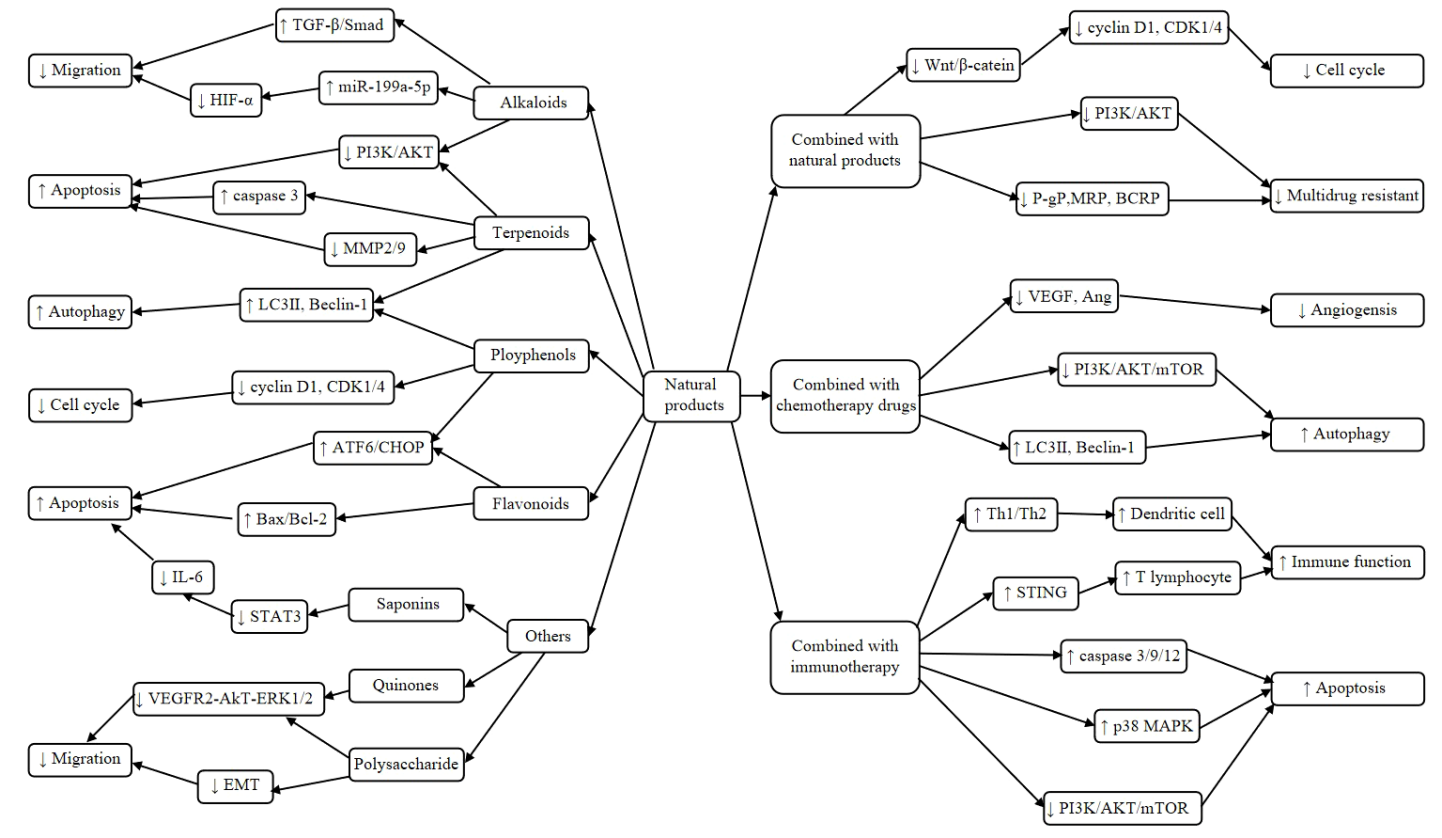
Mechanisms of natural products and combination therapy against liver cancer. Alkaloids could inhibits migration through up-regulating TGF-β/Smad and miR-199a-5p, and down-regulating HIF-α expression. Terpeniods promotes apoptosis by inhibiting the PI3K/AKT pathway and the expression of caspase3. It also induces autophagy through up-regulating the expression of LC3II and Beclin-1. Ployphenols inhibits Cyclin D1 and CDK1/4 expression, which in turn block the cell cycle. It also induces apoptosis by activating ATF6/CHOP signaling pathway. Flavonoids induces apoptosis through activation of the ATF6/CHOP pathway and upregulation the ratio of Bax/Bcl-2. Moreover, other natural products inhibit migration through down-regulating VEGFR2-AKT-ERK1/2 signaling pathway and suppressing EMT. Combination of Natural product ingredients inhibits Cyclin D1 and CDK1/4 expression through downregulation of the Wnt/β-catenin pathway, which in turn inhibits cell proliferation. It also reverses multidrug resistant by decreasing the expression of P-gP, MRP and BCRP. Natural product in combination with chemotherapy suppress angiogensis through down-regulating VEGF and Ang expression. It also induces autophagy by upregulating expression of LC3ii and Beclin-1. Natural product in combination with immunotherapy act by inducing antitumor effects through restoring immune recognition and immune elimination by regulating the immune function. It also induces apoptosis by inhibiting the PI3K/AKT and p38MAPK pathway, which in turn induces apoptosis.

**Figure 3:**
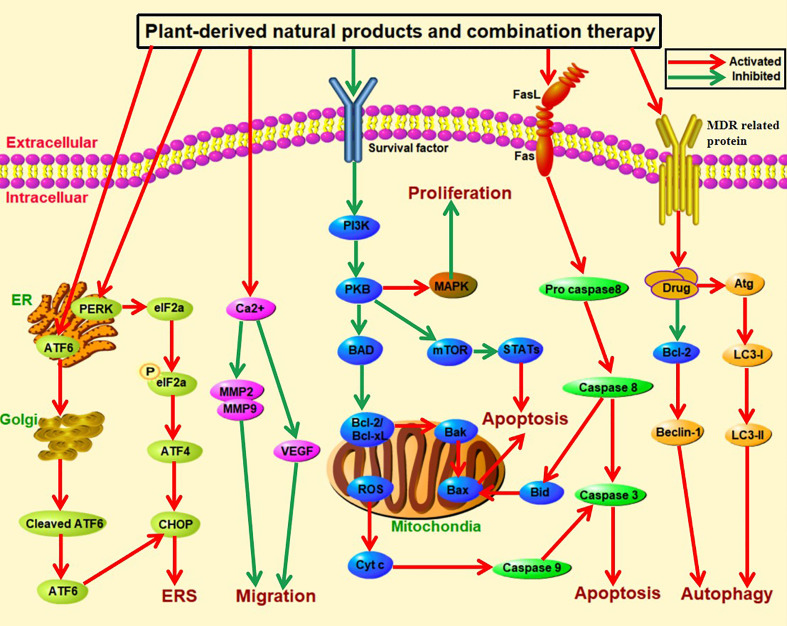
Overview of signaling pathways against liver cancer. Plant-derived natural products and different combination therapy can achieve practical anti-liver-cancer effects through multiple mechanisms, including activation of continuing endoplasmic reticulum stress, suppression of migration, inhibition of cell proliferation, induction of apoptosis and autophagy. For example, it could promote apoptosis through the heavy and continuing endoplasmic reticulum stress; and it coula promote the expression of ATF6, PERK, eIF2α, p-eIF2α, ATF4, CHOP. It also could suppress migration through increasing the concentration of intracellular calciumions, reduce transcriptional activities of MMP-2 and MMP9 and VEGF to inhibit metastasis. Besides, it could increase the expression of Bcl-x; downregulating the expression of Bcl-xL and Bcl-2; suppressing the PI3K/AKT/mTOR signaling pathway and caspase cascade reaction. In the next place, combination therapy could reduce cancer cell proliferation by binding PI3K and subsequently suppressing PKB function and regulating MAPK signaling pathway. It also coula induce apoptosis through activation of STAT3 *via* inhibiting the phosphorylation of mTOR. Moreover, it could promote aopotosis through caspase cascade reactor and. In addition, autophagy is also related to occurrence and development of tumor, natural plants target intracellular autophagy including Beclin-1, PI3K/AKT/mTOR, have been known to control cell proliferation and apoptosis.

The high mortality and morbidity of liver cancer remain a primary challenge for scientific research. Although traditional therapies, such as surgical resection, radiotherapy and chemotherapy, are effective for liver cancer at early stage, the five-year survival rate of liver cancer patients is meager and often ends in failure. In recent years, plant derived natural products and their secondary metabolites possess characteristics of abundant products, low toxicity and side effects, diverse biologic activities, and high content of active ingredients. It has been considered as the most promising candidates for oncology therapies. However, due to poor water solubility, difficult extraction, and easy to develop drug resistance, a single treatment may not be able to achieve satisfactory efficacy. Therapies that combine natural products with chemotherapy drugs or immunotherapy have been developed to target a variety of cancer pathways. The dose requirement of each agent in combination therapy can be reduced, which reduces the side-effects compared to monotherapy. Targeting various pathways *via* multiple drug combinations can control disease preferably and decrease the chance of cancer cells becoming increasingly malignant and incurable. It can also target the heterogeneity of the tumor by two or more pathways to toxicity of cancer cells and disruption of their homeostasis (235). This review mainly focused on the mechanisms of plant-derived natural products and combination therapy against liver cancer. We have listed the mainly compounds and combination therapy and individually summarized their antitumour effects and mechanisms (**Figure 2**). Like many other cancers, liver cancer arises as a result of accumulation of multiple genetic mutations that cause abnormal cellular proliferation. Our study reviewed natural products and different combination therapy that have antitumor effects on liver cancer, which showed possible benefits in treating patients with liver cancer through mechanisms, such as activation of continuing endoplasmic reticulum stress, suppression of migration, inhibition of cell proliferation, induction of apoptosis and autophagy (**Figure 3**). Therefore, it could provide effectively alternative or adjuvant treatment strategies for cancer patients.

However, it is important to note that the study also revealed some disadvantages of combination therapy for cancer. Multiple drug combinations can synergistically increase efficacy, but may also produce unnecessary side effects (236). The target molecular mechanisms of most natural products have not been fully elucidated, especially those related to combinations. Researchers need to further systematically and comprehensively study the immunity or molecular pathology of liver cancer to clarify the relevant mechanisms. Meanwhile, there is still a considerable long way in developing the antitumor strategy of combining natural products with other methods.

## Author contributions

YW: Conceptualization, Data curation, Formal analysis, Investigation, Writing-original draft, Visualization. JL: Writing-review & editing, Supervision. LX: Writing-review & editing, Supervision. All authors contributed to the article and approved the submitted version.
