# Supplementary material for: Plant-derived natural products and combination therapy in liver cancer
Source: Front Oncol. 2023 Feb 14;13:1116532. doi: 10.3389/fonc.2023.1116532 (PMC9971944; doi:10.3389/fonc.2023.1116532)
Supplement: Supplementary file 1 [file Table_1.doc]

**Supplementary Table 1.** Effect and mechanism of plant-derived natural productions on liver cancers.

| **Chemical Family** | **Molecule Name** | | | **Cell Line/ model/patient** | **Mechanism** | **Reference** |
| --- | --- | --- | --- | --- | --- | --- |
| Alkaloids | Matrine | | | HepG2 | Increased Bax/Bcl-2 ratio and inhibited the ERK1/2 signaling pathway | ([18](../../../../C:/Users/%E6%88%B4%E5%B0%94/Desktop/%E7%8E%8B%E7%8E%89%E7%90%B4%E6%96%87%E7%AB%A0/Yuqin%20Wang%2020230131.docx#A18)) |
| Bel7402 SMMC-7721 | Upregulated the expression of miR-199a-5p and reduced the levels of HIF-1α | ([19](../../../../C:/Users/%E6%88%B4%E5%B0%94/Desktop/%E7%8E%8B%E7%8E%89%E7%90%B4%E6%96%87%E7%AB%A0/Yuqin%20Wang%2020230131.docx#A19)) |
| Huh-7  HCCLM3 | Increased the expression of miR-345-5p and reduced the levels of circ0027345 and HOXD3 | ([20](../../../../C:/Users/%E6%88%B4%E5%B0%94/Desktop/%E7%8E%8B%E7%8E%89%E7%90%B4%E6%96%87%E7%AB%A0/Yuqin%20Wang%2020230131.docx#A20)) |
| Orthotopic liver cancer mouse models | Decreased lung metastasis in orthotopic liver cancer mouse models | ([21](../../../../C:/Users/%E6%88%B4%E5%B0%94/Desktop/%E7%8E%8B%E7%8E%89%E7%90%B4%E6%96%87%E7%AB%A0/Yuqin%20Wang%2020230131.docx#A199)) |
| Berberine | | | HepG2  MHCC97-L | Activated Beclin-1 via enhancing p38 MAPK signaling and inhibited Akt activity | ([22](../../../../C:/Users/%E6%88%B4%E5%B0%94/Desktop/%E7%8E%8B%E7%8E%89%E7%90%B4%E6%96%87%E7%AB%A0/Yuqin%20Wang%2020230131.docx#A21)) |
| HepG2 SMMC7721 | Decreased the expression of CD147 in a dose-and time-dependent manner | ([23](../../../../C:/Users/%E6%88%B4%E5%B0%94/Desktop/%E7%8E%8B%E7%8E%89%E7%90%B4%E6%96%87%E7%AB%A0/Yuqin%20Wang%2020230131.docx#A22)) |
| HepG2 | Inhibited TGF-β/Smad signaling pathway through inducing TGF-β1 and interfering EMT | ([24](../../../../C:/Users/%E6%88%B4%E5%B0%94/Desktop/%E7%8E%8B%E7%8E%89%E7%90%B4%E6%96%87%E7%AB%A0/Yuqin%20Wang%2020230131.docx#A23)) |
| Evodiamine | | | HepG2  PLHC-1 | Inactivated PI3K signaling pathway, downregulated Bcl-2 expression and upregulated Bax | ([25](../../../../C:/Users/%E6%88%B4%E5%B0%94/Desktop/%E7%8E%8B%E7%8E%89%E7%90%B4%E6%96%87%E7%AB%A0/Yuqin%20Wang%2020230131.docx#A24)) |
| HepG2  Bel-7402 | Activated Hippo-Yes-associated protein signaling pathway *in vitro* and *in vivo* | ([26](../../../../C:/Users/%E6%88%B4%E5%B0%94/Desktop/%E7%8E%8B%E7%8E%89%E7%90%B4%E6%96%87%E7%AB%A0/Yuqin%20Wang%2020230131.docx#A25)) |
| **Chemical Family** | **Molecule Name** | | | **Cell Line/ model/patient** | **Mechanism** | **Reference** |
| Alkaloids | Camptothecin | | | Huh7  H22 | Upregulated of ROS, Nrf2 and E-cadherin expression; reduced the expression of N-cadherin, MMP9, Snail and Twist; increasing E-cadherin | ([27](../../../../C:/Users/%E6%88%B4%E5%B0%94/Desktop/%E7%8E%8B%E7%8E%89%E7%90%B4%E6%96%87%E7%AB%A0/Yuqin%20Wang%2020230131.docx#A26)) |
| 10-hydroxycamptothecin | | | BEL-7402 | Inhibited proliferation and induce apoptosis of BEL-7402 cell | ([28](../../../../C:/Users/%E6%88%B4%E5%B0%94/Desktop/%E7%8E%8B%E7%8E%89%E7%90%B4%E6%96%87%E7%AB%A0/Yuqin%20Wang%2020230131.docx#A27)) |
| Vincristine | | | Hep3B | Decreased the expression of Ki-67, MMP2, MMP9, increase the expression of cleaved caspase 3 | ([29](../../../../C:/Users/%E6%88%B4%E5%B0%94/Desktop/%E7%8E%8B%E7%8E%89%E7%90%B4%E6%96%87%E7%AB%A0/Yuqin%20Wang%2020230131.docx#A28)) |
| Caffeine | | | HepG2 | Inhibited cell proliferation and induced apoptosis, and blocked the G2/M phase cell cycle | ([30](../../../../C:/Users/%E6%88%B4%E5%B0%94/Desktop/%E7%8E%8B%E7%8E%89%E7%90%B4%E6%96%87%E7%AB%A0/Yuqin%20Wang%2020230131.docx#A29)) |
| Cinchonine | | | HepG2 xenograft mice | Activated caspase-3 and PARP1 cleavage, increased the expression of GRP78 and PERK, and suppressed HepG2 xenograft tumor growth in mice | ([31](../../../../C:/Users/%E6%88%B4%E5%B0%94/Desktop/%E7%8E%8B%E7%8E%89%E7%90%B4%E6%96%87%E7%AB%A0/Yuqin%20Wang%2020230131.docx#A200)) |
| Sanguinarine | | | Liver cancer xenograft model | Induced cell cycle arrest and ROS-associated apoptosis, and suppressed tumor growth in an HCC xenograft model, with little cytotoxicity | ([33](../../../../C:/Users/%E6%88%B4%E5%B0%94/Desktop/%E7%8E%8B%E7%8E%89%E7%90%B4%E6%96%87%E7%AB%A0/Yuqin%20Wang%2020230131.docx#A201)) |
| Terpenoids | | Dsaikosaponin D | | PLC  MHCC-97H  SMMC-7721  Huh7 | Up-regulated the protein expression of LC3Ⅱ and Beclin 1, down-regulated the protein expression of P-S6K1 | ([34](../../../../C:/Users/%E6%88%B4%E5%B0%94/Desktop/%E7%8E%8B%E7%8E%89%E7%90%B4%E6%96%87%E7%AB%A0/Yuqin%20Wang%2020230131.docx#A30)) |
| SMMC-7721  MHCC97L | Increased radiation-induced apoptosis of liver cells via inhibiting mTOR phosphorylation | ([35](../../../../C:/Users/%E6%88%B4%E5%B0%94/Desktop/%E7%8E%8B%E7%8E%89%E7%90%B4%E6%96%87%E7%AB%A0/Yuqin%20Wang%2020230131.docx#A31)) |
| HepG2 | Reduced the expression of Ki67, upregulated cleaved caspase-3 expression | ([36](../../../../C:/Users/%E6%88%B4%E5%B0%94/Desktop/%E7%8E%8B%E7%8E%89%E7%90%B4%E6%96%87%E7%AB%A0/Yuqin%20Wang%2020230131.docx#A32)) |
| **Chemical Family** | | **Molecule Name** | | **Cell Line/ model/patient** | **Mechanism** | **Reference** |
| Terpenoids | | Triptolide | | HepG2 | Up-regulated the expression of miR-194, Bax/Bcl-2 ratio, LC3 and Beclin-1 | ([37](../../../../C:/Users/%E6%88%B4%E5%B0%94/Desktop/%E7%8E%8B%E7%8E%89%E7%90%B4%E6%96%87%E7%AB%A0/Yuqin%20Wang%2020230131.docx#A33)) |
| HepG2 | Downregulated the expression of Ras in the HepG2 cells | ([38](../../../../C:/Users/%E6%88%B4%E5%B0%94/Desktop/%E7%8E%8B%E7%8E%89%E7%90%B4%E6%96%87%E7%AB%A0/Yuqin%20Wang%2020230131.docx#A34)) |
| H22 | Decreased the expression of COX-2 | ([39](../../../../C:/Users/%E6%88%B4%E5%B0%94/Desktop/%E7%8E%8B%E7%8E%89%E7%90%B4%E6%96%87%E7%AB%A0/Yuqin%20Wang%2020230131.docx#A35)) |
| Ginsenoside Rg3 | | SMMC-7721 | Decreased the expression of p-AKT, p-PI3K, MMP2, MMP9 and lncRNA HOTAIR | ([40](../../../../C:/Users/%E6%88%B4%E5%B0%94/Desktop/%E7%8E%8B%E7%8E%89%E7%90%B4%E6%96%87%E7%AB%A0/Yuqin%20Wang%2020230131.docx#A36)) |
| Artemisinin | | Hepa1-6 | Blocked the accumulation and function of MDSCs by regulating PI3K/AKT/mTOR and MAPK signaling pathways; polarizated M2-like pro-tumor phenotype to M1-like anti-tumor phenotype; enhanced the effect of anti-PD-L1 immunotherapy | ([41](../../../../C:/Users/%E6%88%B4%E5%B0%94/Desktop/%E7%8E%8B%E7%8E%89%E7%90%B4%E6%96%87%E7%AB%A0/Yuqin%20Wang%2020230131.docx#A37)) |
| Dihydroartemisinine | | HepG2 | Regulated Bcl-2/Bax/caspase-3 apoptosis signal pathway | ([42](../../../../C:/Users/%E6%88%B4%E5%B0%94/Desktop/%E7%8E%8B%E7%8E%89%E7%90%B4%E6%96%87%E7%AB%A0/Yuqin%20Wang%2020230131.docx#A38)) |
| Huh-7  HepG2 | Promoted the formation of PEBP1/15-LO and lipid peroxidation in cell membrane | ([43](../../../../C:/Users/%E6%88%B4%E5%B0%94/Desktop/%E7%8E%8B%E7%8E%89%E7%90%B4%E6%96%87%E7%AB%A0/Yuqin%20Wang%2020230131.docx#A39)) |
| Celastrol | | HepG2 | Activated AMPK signaling pathway by inhibiting lipid metabolism | ([44](../../../../C:/Users/%E6%88%B4%E5%B0%94/Desktop/%E7%8E%8B%E7%8E%89%E7%90%B4%E6%96%87%E7%AB%A0/Yuqin%20Wang%2020230131.docx#A40)) |
| HepG2  SMMC-7721 | Reduced the expression of NF-κB and TNF-α in a dose and time-dependent manner | ([45](../../../../C:/Users/%E6%88%B4%E5%B0%94/Desktop/%E7%8E%8B%E7%8E%89%E7%90%B4%E6%96%87%E7%AB%A0/Yuqin%20Wang%2020230131.docx#A41)) |
| Norcantharidin | | Huh7 | Activated p53 and p21 proteins and blocked the G2/M phase cell cycle | ([47](../../../../C:/Users/%E6%88%B4%E5%B0%94/Desktop/%E7%8E%8B%E7%8E%89%E7%90%B4%E6%96%87%E7%AB%A0/Yuqin%20Wang%2020230131.docx#A42)) |
| **Chemical Family** | | **Molecule Name** | | **Cell Line/ model/patient** | **Mechanism** | **Reference** |
| Terpenoids | | Genipin | | Orthotopic liver cancer mouse models | Inhibited IRE1a-mediated infiltration and priming of tumour associated macrophages (TAMs), reduced infiltration of inflammatory monocytes into liver and tumour, and inhibited the TAMs migration | ([48](../../../../C:/Users/%E6%88%B4%E5%B0%94/Desktop/%E7%8E%8B%E7%8E%89%E7%90%B4%E6%96%87%E7%AB%A0/Yuqin%20Wang%2020230131.docx#A202)) |
| Andrographolide | | Liver cancer xenograft model | Inhibited the hepatoma tumor growth and alters the expression of miRNAs profile and downstream signals | ([49](../../../../C:/Users/%E6%88%B4%E5%B0%94/Desktop/%E7%8E%8B%E7%8E%89%E7%90%B4%E6%96%87%E7%AB%A0/Yuqin%20Wang%2020230131.docx#A203)) |
| Ployphenols | | Curcumin | | HepG2 | Downregulated the expression of BCLAF1, inhibited the activation of PI3K/AKT/GSK-3β pathway, and triggered mitochondrial apoptosis in liver cancer | ([50](../../../../C:/Users/%E6%88%B4%E5%B0%94/Desktop/%E7%8E%8B%E7%8E%89%E7%90%B4%E6%96%87%E7%AB%A0/Yuqin%20Wang%2020230131.docx#A43)) |
| SMMC-7721 | Reduced the expression of Bcl-2 and increase Cleaved Caspase-3 expression *in vitro*; inhibited SMMC-7721 cell nude mice xenograft tumor growth by activating ERS pathway | ([51](../../../../C:/Users/%E6%88%B4%E5%B0%94/Desktop/%E7%8E%8B%E7%8E%89%E7%90%B4%E6%96%87%E7%AB%A0/Yuqin%20Wang%2020230131.docx#A44)) |
| Huh-7  MHCC-97H  HepG2 | Activated the GSDME-related scorch death and mitochondria-dependent apoptosis signaling pathways | ([52](../../../../C:/Users/%E6%88%B4%E5%B0%94/Desktop/%E7%8E%8B%E7%8E%89%E7%90%B4%E6%96%87%E7%AB%A0/Yuqin%20Wang%2020230131.docx#A45)) |
| Resveratrol | | HepG2  Huh7 | Increased the expression of E-cadherin and miR-186-5p, and decreased the expression of vimentin and Twist1 | ([54](../../../../C:/Users/%E6%88%B4%E5%B0%94/Desktop/%E7%8E%8B%E7%8E%89%E7%90%B4%E6%96%87%E7%AB%A0/Yuqin%20Wang%2020230131.docx#A46)) |
| HepG2  Hep3B | Decreased the expression of MARCH1 and phospho-protein kinase B (p-AKT), increased the expression of PTEN dose-dependently | ([55](../../../../C:/Users/%E6%88%B4%E5%B0%94/Desktop/%E7%8E%8B%E7%8E%89%E7%90%B4%E6%96%87%E7%AB%A0/Yuqin%20Wang%2020230131.docx#A47)) |
| **Chemical Family** | | **Molecule Name** | | **Cell Line/ model/patient** | **Mechanism** | **Reference** |
| Ployphenols | | 6-gingerol | | Huh7 | Regulated the AKT/EER1/2 signaling pathway, increased P27/Kip1 and p21/Cip1 expression and inhibited cyclins D1, c-myc and CDK4 protein expression | ([56](../../../../C:/Users/%E6%88%B4%E5%B0%94/Desktop/%E7%8E%8B%E7%8E%89%E7%90%B4%E6%96%87%E7%AB%A0/Yuqin%20Wang%2020230131.docx#A48)) |
| Epigallocatechin gallate | | SMMC-7721  HepG2 | Involved with the signaling intervention of MAPK/ERK1/2 and PI3K/AKT/HIF-1α/VEGF pathways | ([57](../../../../C:/Users/%E6%88%B4%E5%B0%94/Desktop/%E7%8E%8B%E7%8E%89%E7%90%B4%E6%96%87%E7%AB%A0/Yuqin%20Wang%2020230131.docx#A49)) |
| HepG2 | Inhibited the proliferation of HepG2 cells and the tumor growth in bearing cancer nude mice | ([58](../../../../C:/Users/%E6%88%B4%E5%B0%94/Desktop/%E7%8E%8B%E7%8E%89%E7%90%B4%E6%96%87%E7%AB%A0/Yuqin%20Wang%2020230131.docx#A50)) |
| BEL-7404/ADR | Induced apoptosis and arrested cells in S/G2 phase of the cell cycle in a dose-dependent manner | ([59](../../../../C:/Users/%E6%88%B4%E5%B0%94/Desktop/%E7%8E%8B%E7%8E%89%E7%90%B4%E6%96%87%E7%AB%A0/Yuqin%20Wang%2020230131.docx#A51)) |
| Tannic acid | | HepG2 | Induced apoptosis by DNA disruption and oxidative stress, mediated caspase-dependent and non-caspase-dependent mechanisms | ([60](../../../../C:/Users/%E6%88%B4%E5%B0%94/Desktop/%E7%8E%8B%E7%8E%89%E7%90%B4%E6%96%87%E7%AB%A0/Yuqin%20Wang%2020230131.docx#A52)) |
| HepG2 | Enhanced the activation level of endoplasmic reticulum stress ATF6-CHOP pathway | ([61](../../../../C:/Users/%E6%88%B4%E5%B0%94/Desktop/%E7%8E%8B%E7%8E%89%E7%90%B4%E6%96%87%E7%AB%A0/Yuqin%20Wang%2020230131.docx#A53)) |
| Magnolol | | SK-Hep1/luc2 HCC bearing animal model | Decreased the expression of p-ERK, NF-κB p65 (Ser536), MMP-9, VEGF, XIAP, and CyclinD1, and increased the expression of caspase-8 and caspase-9 | ([62](../../../../C:/Users/%E6%88%B4%E5%B0%94/Desktop/%E7%8E%8B%E7%8E%89%E7%90%B4%E6%96%87%E7%AB%A0/Yuqin%20Wang%2020230131.docx#A204)) |
| Rosmarinic acid | | H22 tumor-bearing mice | Decreased the expressions of IL-6, IL-10 and signal transducer and activator of transcription 3, up-regulated Bax, caspase-3 and down-regulate Bcl-2 | ([63](../../../../C:/Users/%E6%88%B4%E5%B0%94/Desktop/%E7%8E%8B%E7%8E%89%E7%90%B4%E6%96%87%E7%AB%A0/Yuqin%20Wang%2020230131.docx#A205)) |
| **Chemical Family** | | **Molecule Name** | | **Cell Line/ model/patient** | **Mechanism** | **Reference** |
| Flavonoids | | Kaempferol | | HepG2 | Decreased the expression of miR-21 and increased the expression of PTEN, and inactivated PI3K/AKT/mTOR signaling pathway | ([64](../../../../C:/Users/%E6%88%B4%E5%B0%94/Desktop/%E7%8E%8B%E7%8E%89%E7%90%B4%E6%96%87%E7%AB%A0/Yuqin%20Wang%2020230131.docx#A55)) |
| HepG2 | Decreased the expression of CDK1 and increased the expression of Bax and JUN | ([65](../../../../C:/Users/%E6%88%B4%E5%B0%94/Desktop/%E7%8E%8B%E7%8E%89%E7%90%B4%E6%96%87%E7%AB%A0/Yuqin%20Wang%2020230131.docx#A56)) |
| HepG2 | Increased the protein expression of Bax, GRP78, GRP94, PERK, Cleaved ATF6, IRE1α, CHOP, caspase3 and caspase4 | ([66](../../../../C:/Users/%E6%88%B4%E5%B0%94/Desktop/%E7%8E%8B%E7%8E%89%E7%90%B4%E6%96%87%E7%AB%A0/Yuqin%20Wang%2020230131.docx#A57)) |
| Silymarin | | MHCC97 | Reduced Akt phosphorylation by decreasing integrinβ1, VEGF and MMP-9; restored the cell adhesion ability by upregulating E-cadherin | ([67](../../../../C:/Users/%E6%88%B4%E5%B0%94/Desktop/%E7%8E%8B%E7%8E%89%E7%90%B4%E6%96%87%E7%AB%A0/Yuqin%20Wang%2020230131.docx#A58)) |
| Silibinin | | HepG2 | Induced apoptosis, suppressed angiogenesis and blocked cell cycle | ([69](../../../../C:/Users/%E6%88%B4%E5%B0%94/Desktop/%E7%8E%8B%E7%8E%89%E7%90%B4%E6%96%87%E7%AB%A0/Yuqin%20Wang%2020230131.docx#A60)) |
| HepG2 | Inhibited the proliferative capacity of the cells as well as migration | ([70](../../../../C:/Users/%E6%88%B4%E5%B0%94/Desktop/%E7%8E%8B%E7%8E%89%E7%90%B4%E6%96%87%E7%AB%A0/Yuqin%20Wang%2020230131.docx#A61)) |
| Quercetin | | Liver cancer rats | Reduced the expression of cancer stem cells and myofibroblasts markers, as well as the expression of ABCC3 | ([72](../../../../C:/Users/%E6%88%B4%E5%B0%94/Desktop/%E7%8E%8B%E7%8E%89%E7%90%B4%E6%96%87%E7%AB%A0/Yuqin%20Wang%2020230131.docx#A63)) |
| HepG2 | Inhibited the proliferation *in vitro* and *in vivo* and caused tumor cells apoptosis | ([73](../../../../C:/Users/%E6%88%B4%E5%B0%94/Desktop/%E7%8E%8B%E7%8E%89%E7%90%B4%E6%96%87%E7%AB%A0/Yuqin%20Wang%2020230131.docx#A64)) |
| HepG2 | Increased the expression of p-JNK, p-AKT, p-p38, caspase3 and decreased the expression of Bcl-xL, p-ERK and NF-κB | ([74](../../../../C:/Users/%E6%88%B4%E5%B0%94/Desktop/%E7%8E%8B%E7%8E%89%E7%90%B4%E6%96%87%E7%AB%A0/Yuqin%20Wang%2020230131.docx#A65)) |
| **Chemical Family** | | **Molecule Name** | | **Cell Line/ model/patient** | **Mechanism** | **Reference** |
| Flavonoids | | Chrysin | | SMMC-7721 | Inhibited proliferation and induced apoptosis by activating MAPK signaling molecules | ([76](../../../../C:/Users/%E6%88%B4%E5%B0%94/Desktop/%E7%8E%8B%E7%8E%89%E7%90%B4%E6%96%87%E7%AB%A0/Yuqin%20Wang%2020230131.docx#A67)) |
| H22 | Reduced the expression of PD-L1 through blocking of STAT3 and NF-κB pathways | ([77](../../../../C:/Users/%E6%88%B4%E5%B0%94/Desktop/%E7%8E%8B%E7%8E%89%E7%90%B4%E6%96%87%E7%AB%A0/Yuqin%20Wang%2020230131.docx#A68)) |
| Diosmetin | | SK-HEP-1  MHcc97H | Regulated the PKCδ/MAPK/ matrix metallo proteinase pathway | ([78](../../../../C:/Users/%E6%88%B4%E5%B0%94/Desktop/%E7%8E%8B%E7%8E%89%E7%90%B4%E6%96%87%E7%AB%A0/Yuqin%20Wang%2020230131.docx#A69)) |
| HepG2 | Downregulated Bcl-2, cdc2, cyclinB1, and up-regulated Bax, Cleaved-caspase3, Cleaved-caspase8, Cleaved-PARP, Bak, p53, p21 | ([79](../../../../C:/Users/%E6%88%B4%E5%B0%94/Desktop/%E7%8E%8B%E7%8E%89%E7%90%B4%E6%96%87%E7%AB%A0/Yuqin%20Wang%2020230131.docx#A70)) |
| Hep3B  HCCLM3 | Reduced the expression of BCL2, CDK1 and CCND1 in a dose-dependent manner *in vitro*, with reducing the expression of proliferation marker Ki67 *in vivo* | ([80](../../../../C:/Users/%E6%88%B4%E5%B0%94/Desktop/%E7%8E%8B%E7%8E%89%E7%90%B4%E6%96%87%E7%AB%A0/Yuqin%20Wang%2020230131.docx#A71)) |
| Baicalin | | Orthotopic HCC implantation model | Induced repolarisation of TAM and M2-like macrophages, which is associated with elevated autophagy, and transcriptional activation of RelB/p52 pathway | ([81](../../../../C:/Users/%E6%88%B4%E5%B0%94/Desktop/%E7%8E%8B%E7%8E%89%E7%90%B4%E6%96%87%E7%AB%A0/Yuqin%20Wang%2020230131.docx#A206)) |
| Quinones | | Emodin | | HepG2 | Regulated VEGFR2-AkT-ERK1/2 signaling pathway and miR-34a signaling pathway | ([82](../../../../C:/Users/%E6%88%B4%E5%B0%94/Desktop/%E7%8E%8B%E7%8E%89%E7%90%B4%E6%96%87%E7%AB%A0/Yuqin%20Wang%2020230131.docx#A72)) |
| Aloin | | MHCC97H | Inhibited proliferation, migration and invasion and induced apoptosis through regulating Bcl-2 /Bax expression | ([83](../../../../C:/Users/%E6%88%B4%E5%B0%94/Desktop/%E7%8E%8B%E7%8E%89%E7%90%B4%E6%96%87%E7%AB%A0/Yuqin%20Wang%2020230131.docx#A73)) |
| **Chemical Family** | | | **Molecule Name** | **Cell Line/ model/patient** | **Mechanism** | **Reference** |
| Quinones | | | Tanshinone | HepG2  Huh7 | Induced G0/G1 phase cell cycle arrest via decreasing the expression of cyclin D1, and increasing p21, as well as mediated the inhibition of p53/damage-regulated autophagy and induced apoptosis | ([84](../../../../C:/Users/%E6%88%B4%E5%B0%94/Desktop/%E7%8E%8B%E7%8E%89%E7%90%B4%E6%96%87%E7%AB%A0/Yuqin%20Wang%2020230131.docx#A74)) |
| Cryptotanshinone | HepG2 | Increased the accumulation of ROS by inhibiting the expression of xCT and GPX4 to induce the ferroptosis | ([85](../../../../C:/Users/%E6%88%B4%E5%B0%94/Desktop/%E7%8E%8B%E7%8E%89%E7%90%B4%E6%96%87%E7%AB%A0/Yuqin%20Wang%2020230131.docx#A75)) |
| Polysaccharide | | | Astragalus polysaccharides | HepG2 | Inhibited proliferation, reversed the drug resistance of 5-fluorouracil, inhibited EMT and induced apoptosis pathway | ([87](../../../../C:/Users/%E6%88%B4%E5%B0%94/Desktop/%E7%8E%8B%E7%8E%89%E7%90%B4%E6%96%87%E7%AB%A0/Yuqin%20Wang%2020230131.docx#A76), [88](../../../../C:/Users/%E6%88%B4%E5%B0%94/Desktop/%E7%8E%8B%E7%8E%89%E7%90%B4%E6%96%87%E7%AB%A0/Yuqin%20Wang%2020230131.docx#A77), [89](../../../../C:/Users/%E6%88%B4%E5%B0%94/Desktop/%E7%8E%8B%E7%8E%89%E7%90%B4%E6%96%87%E7%AB%A0/Yuqin%20Wang%2020230131.docx#A78), [90](../../../../C:/Users/%E6%88%B4%E5%B0%94/Desktop/%E7%8E%8B%E7%8E%89%E7%90%B4%E6%96%87%E7%AB%A0/Yuqin%20Wang%2020230131.docx#A79)) |
| HepG2.215 | Blocked HepG2.215 cells from entering the G2/M phase, activated the apoptosis system and induce apoptosis | ([91](../../../../C:/Users/%E6%88%B4%E5%B0%94/Desktop/%E7%8E%8B%E7%8E%89%E7%90%B4%E6%96%87%E7%AB%A0/Yuqin%20Wang%2020230131.docx#A80)) |
| Poria cocos polysaccharide | HepG2 | Inhibited the expression of NLRP3/caspase-1/GSDMD in the classical pyroptosis pathway | ([92](../../../../C:/Users/%E6%88%B4%E5%B0%94/Desktop/%E7%8E%8B%E7%8E%89%E7%90%B4%E6%96%87%E7%AB%A0/Yuqin%20Wang%2020230131.docx#A81)) |
| Lycium barbarum polysaccharide | SMMC-7721 | Reduced the expression of MMP-2, MMP-9 and VEGF | ([93](../../../../C:/Users/%E6%88%B4%E5%B0%94/Desktop/%E7%8E%8B%E7%8E%89%E7%90%B4%E6%96%87%E7%AB%A0/Yuqin%20Wang%2020230131.docx#A82)) |
| Saponins | | | Astragalus saponins | H22 tumor-bearing mice | Up-regulated the expression of caspase3, inhibited the expression of interleukin-6 by inhibiting the STAT3 signaling pathway | ([94](../../../../C:/Users/%E6%88%B4%E5%B0%94/Desktop/%E7%8E%8B%E7%8E%89%E7%90%B4%E6%96%87%E7%AB%A0/Yuqin%20Wang%2020230131.docx#A83)) |
| HepG2 | Regulated oxidative stress and NF-κB signaling pathway | ([95](../../../../C:/Users/%E6%88%B4%E5%B0%94/Desktop/%E7%8E%8B%E7%8E%89%E7%90%B4%E6%96%87%E7%AB%A0/Yuqin%20Wang%2020230131.docx#A84)) |
| **Chemical Family** | | | **Molecule Name** | **Cell Line/ model/patient** | **Mechanism** | **Reference** |
| Saponins | | | Astragalus saponins | H22 tumor-bearing mice | Regulated oxidative stress and NF-κB signaling pathway, promoted the secretion of interleukin-2 by improving the proliferation and transformation of lymphocytes, improved the immune function of rats | ([96](../../../../C:/Users/%E6%88%B4%E5%B0%94/Desktop/%E7%8E%8B%E7%8E%89%E7%90%B4%E6%96%87%E7%AB%A0/Yuqin%20Wang%2020230131.docx#A85)) |
| Compounds | | | Huangqi Sijunzi decoction | Primary liver cancer patient | Promoted postoperative recovery of the liver function and immunologic function of the patients undergoing primary liver cancer operation, alleviated clinical symptoms and facilitated postoperative recovery of the patients | ([97](../../../../C:/Users/%E6%88%B4%E5%B0%94/Desktop/%E7%8E%8B%E7%8E%89%E7%90%B4%E6%96%87%E7%AB%A0/Yuqin%20Wang%2020230131.docx#A207)) |
| Yiqi Huoxue Fuzheng Jiedu detoxification | Advanced liver cancer patient | Improved its clinical symptoms and liver function | ([98](../../../../C:/Users/%E6%88%B4%E5%B0%94/Desktop/%E7%8E%8B%E7%8E%89%E7%90%B4%E6%96%87%E7%AB%A0/Yuqin%20Wang%2020230131.docx#A208)) |
| Gleditsiae Spina ointment | Primary liver cancer patient | Reduced the dose of opioid painkillers and the number of times of breakthrough pain and improved patients' quality of life | ([99](../../../../C:/Users/%E6%88%B4%E5%B0%94/Desktop/%E7%8E%8B%E7%8E%89%E7%90%B4%E6%96%87%E7%AB%A0/Yuqin%20Wang%2020230131.docx#A209)) |
| Xingqi Sanjie Huayu Prescription | Primary liver cancer patient | Improved the clinical symptoms, reduced the levels of serum tumor markers, enhanced the immune function and liver function, and reduced the incidence of adverse reactions | ([100](../../../../C:/Users/%E6%88%B4%E5%B0%94/Desktop/%E7%8E%8B%E7%8E%89%E7%90%B4%E6%96%87%E7%AB%A0/Yuqin%20Wang%2020230131.docx#A210)) |
| Triple therapy | Primary liver cancer patient | Improved patients’ clinical syndrome and quality of life, promoted the improvement of liver function, regulated immune function，reduce AFP level | ([101](../../../../C:/Users/%E6%88%B4%E5%B0%94/Desktop/%E7%8E%8B%E7%8E%89%E7%90%B4%E6%96%87%E7%AB%A0/Yuqin%20Wang%2020230131.docx#A211)) |
| Fuzheng Huayu Jiedu prescription | Advanced liver cancer patient | Reduced the levels of inflammatory factors, inhibited inflammatory responses, improved immune function and enhanced resistance | ([102](../../../../C:/Users/%E6%88%B4%E5%B0%94/Desktop/%E7%8E%8B%E7%8E%89%E7%90%B4%E6%96%87%E7%AB%A0/Yuqin%20Wang%2020230131.docx#A212)) |
| Hedyotis diffusa injection | Primary liver cancer patient | Improved the disease control rate and improved the levels of T cell subsets | ([103](../../../../C:/Users/%E6%88%B4%E5%B0%94/Desktop/%E7%8E%8B%E7%8E%89%E7%90%B4%E6%96%87%E7%AB%A0/Yuqin%20Wang%2020230131.docx#A213)) |
